# *Salmonella* exploits a quorum-sensing family signal of the gut commensal *Stenotrophomonas maltophilia* to facilitate its colonization

**DOI:** 10.1080/19490976.2026.2699455

**Published:** 2026-07-08

**Authors:** Rimi Chowdhury, Erick M. Bosire, Lindsay R. Wolverton, Paulina D. Pavinski Bitar, Katherine E. Bell, Ivan Keresztes, Rory C. Chien, Craig Altier

**Affiliations:** a Department of Microbiology, College of Arts and Science, Miami University, Oxford, OH, USA; b Department of Population Medicine and Diagnostic Sciences, College of Veterinary Medicine, Cornell University, Ithaca, NY, USA; c Department of Chemistry and Chemical Biology, Cornell University, Ithaca, NY, USA

**Keywords:** Host pathogen interaction, *Salmonella*, quorum sensing signals, microbiome, *Stenotrophomonas maltophilia*, *Salmonella* virulence, host microbe interactions

## Abstract

Successful colonization by enteric pathogens requires overcoming colonization resistance of the native microbiota while tightly regulating the expression of energetically-expensive virulence factors. Here we describe a feedback mechanism by which the enteric pathogen *Salmonella* orchestrates this balance through environmental manipulation. We show that *Salmonella*-induced oxidative stress can stimulate the colonic resident *Stenotrophomonas maltophilia* to enhance the secretion of the diffusible signal factor *cis*-2-hexadecenoic acid (c2-HDA), a potent repressor of *Salmonella* virulence. By sensing this metabolite, *Salmonella* can attenuate its own virulence program to favor proliferation and colonic colonization. In murine models, *Salmonella* colonization was significantly enhanced in the colon, and inflammation reduced, in the presence of c2-HDA produced by *S. maltophilia*. Moreover, the ability of *Salmonella* to recognize c2-HDA within the murine colon was crucial for its successful colonization. These findings reveal a pathogen-commensal signaling axis through which pathogen-driven inflammatory cues reshape the metabolic output of the microbiota, generating regulatory signals that are co-opted to optimize pathogen fitness in the gut.

## Highlights

• *Salmonella*-driven inflammation can augment the production of a diffusible signal factor by the gut-associated organism *Stenotrophomonas maltophilia.*
• Oxidative stress prompts *S. maltophilia* to secrete c2-HDA, a diffusible fatty acid signal that represses *Salmonella* virulence.
• c2-HDA attenuates *Salmonella* invasion gene expression, enabling the transition to a proliferative state.
• *Salmonella* senses this microbiota-derived metabolite to fine-tune virulence and enhance colonic colonization.
• Findings uncover a pathogen-host-microbiota-pathogen feedback signaling loop integrating host responses, microbiota metabolism, and pathogen fitness.

## Introduction

Enteric pathogens confront a formidable but essential task: gaining and maintaining colonization within a host. For successful infection, these pathogens must express their virulence factors, which is an energetically expensive process.^1-6^ They must, however, simultaneously overcome colonization resistance, by which the vast native microbiota that occupy existing spatial and nutritional niches starve the pathogens of essential resources.^7-9^ To overcome these obstacles, enteric pathogens such as *Salmonella* have evolved a finely tuned strategy that couples environmental sensing with dynamic regulation of its virulence program. By employing environmental compounds as signals, *Salmonella* initiates its full virulence program, which triggers acute mucosal inflammation, thereby disrupting the microbiota and releasing nutrient pools and electron acceptors that support *Salmonella* expansion.^10-14^ Subsequently, in a spatially and temporally regulated manner, the pathogen downregulates its virulence gene expression and transitions to a proliferative mode, maximizing resource acquisition and transmission potential.^15-20^

*Salmonella* has thus evolved the ability to sense and respond to chemical cues along its journey through the intestine. Within the duodenum, bile represses *Salmonella* functions required for its penetration of the intestinal epithelium,^21^ ^,^ ^22^ thus preventing induction of inflammation while in the proximal portion of the small intestine. In the ileum, *Salmonella* encounters formate in high concentration, which serves as a signal to activate invasion genes, thus making the ileum the preferred region for *Salmonella* invasion.^12^ ^,^ ^23^ As the pathogen progresses into the colon, the chemical landscape shifts again. Here, *Salmonella* encounters an abundance of short- and long-chain fatty acids, many of which have been shown to repress invasion gene expression.^15-18^ ^,^ ^23^ This spatial regulation ensures that energy-expensive invasion mechanisms are activated only in the regions appropriate to elicit a robust inflammatory response, maximizing infection efficiency while minimizing metabolic burden.

Central to virulence regulation is HilD, a master transcriptional activator that facilitates the expression of the macromolecular syringe-like apparatus called the Type 3 Secretion System-1 (T3SS-1) via a feedforward regulatory loop involving *hilA*, *hilC*, and *rtsA.* ^24-27^ Under optimal conditions, HilD binds DNA operator regions of these regulators to initiate a transcriptional cascade, culminating in the production of the T3SS-1. This process is, however, exquisitely sensitive to environmental signals. Several intestinal compounds have been shown to disrupt the DNA-binding ability of HilD and to mediate its rapid degradation.^15^ ^,^ ^17-19^ ^,^ ^28^ Hence, by this mechanism *Salmonella* can utilize environmental compounds to efficiently repress its virulence functions.

Several intestinal compounds are known to be utilized by *Salmonella* for virulence regulation; prominent amongst these are fatty acids.^29^ ^,^ ^30^ In particular, propionic acid, oleic acid and *cis*-2 hexadecenoic acid (c2-HDA), all found in the colon, have been shown to directly target HilD and prevent invasion gene expression by mediating its degradation and disrupting its ability to bind to promoter DNA^15^ ^,^ ^17^ ^,^ ^27^. c2-HDA is the most potent fatty-acid repressor, reducing pathogen virulence at micromolar concentrations not only in *Salmonella* but also in the enteric pathogens *Shigella flexneri* ^20^ ^,^ ^31^ and *Vibrio cholerae.* ^32^ Belonging to the Diffusible Signal Factor (DSF) family, c2-HDA features a *cis*-2 unsaturation, a structural hallmark of quorum-sensing molecules originally identified in the plant pathogen *Xylella fastidiosa.* ^33-35^ DSFs are synthesized by the *rpfF*-encoded dehydratase/thioesterase that converts fatty acyl-CoA molecules to *cis*-2 unsaturated fatty acids.^36^ ^,^ ^37^ Although various gut-associated genera such as *Burkholderia*, *Cronobacter*, and *Stenotrophomonas* possess *rpfF*, none has been confirmed to produce c2-HDA.^38^ ^,^ ^39^ As c2-HDA has been isolated from the murine colon,^19^ the presence of one or more DSF-producing organisms amongst the microbiota appears likely.

Here we uncover a novel interplay between pathogen-induced inflammation and the gut microbiota that is utilized by *Salmonella* to regulate its lifecycle within an animal host. We show that *S. maltophilia*, a resident of the colonic crypts^40^ ^,^ ^41^ produces c2-HDA in an *rpfF*-dependent manner in response to oxidative stress, such as that generated by macrophage-derived reactive oxygen and nitrogen species during inflammation. This reveals an unexpected consequence of the host response: It prompts a commensal microbe to enhance the production of a metabolite that is utilized by *Salmonella* to suppress virulence. Moreover, we demonstrate that *Salmonella* benefits from this dynamic: inflammation triggered by *Salmonella* creates an environment that amplifies production by *S. maltophilia* of virulence-repressing compounds. These compounds, in turn, are utilized to downregulate *Salmonella's* own invasion genes, facilitating its transition from an invasive to a colonizing lifestyle. This study thus reveals an instance of pathogen-host-microbe-pathogen crosstalk by which *Salmonella* utilizes microbial products elicited by the host inflammatory response to optimize its colonization strategy. More broadly, it demonstrates that pathogen success in the gut is not simply a battle between host and invader, but a sophisticated balance involving the triad of chemical signaling, microbial ecology, and host response.

## Results

### Fatty acid signals regulate the *in vivo* balance between *Salmonella* virulence and proliferation

**Figure 1.**
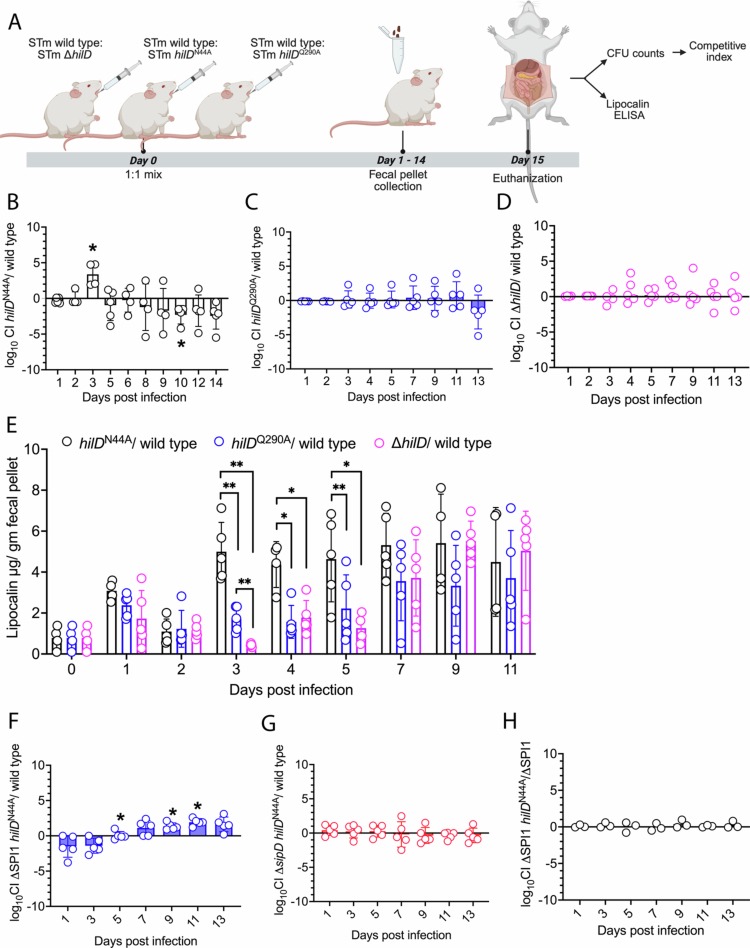
Fatty acid signals regulate the *in vivo* balance between *Salmonella* virulence and proliferation. (a). Schematic diagram of the competitive index determination using the 129X1/SvJ mouse model, made in BioRender. (b–d) and (f–h). Competitive indices for the wild type vs (b) *Salmonella* (STm) *hilD* ^N44A^, (c) *hilD* ^Q290A^, (d) Δ*hilD* control, (f) ΔSPI1, *hilD* ^N44A^, (g) Δ*sipD, hilD* ^N44A^; and (h) ΔSPI1, *hilD* ^N44A^ versus ΔSPI1. Fecal pellets were collected at indicated days post infection and competitive indices were calculated from CFU counts. Differences in CFU between the mutants and wild type were calculated using the Mann–Whitney test. *p < 0.05. (e). Quantification of colonic inflammation in fecal pellets collected at indicated days post infection by fecal lipocalin ELISA. Bars show mean ± SD of five biological replicates. Differences between the indicated groups were calculated by Mann–Whitney test. **p < 0.01, *p < 0.05.

*Salmonella* utilizes a set of virulence factors to overcome host- and microbiota-mediated colonization resistance and establish a niche in the gut.^42^ As expression of these virulence factors often comes with a fitness cost, *Salmonella* integrates gut chemical signals to ensure that these factors are expressed only at permissible sites, and that they are repressed when not needed.^16-19^ ^,^ ^22^ We and others have previously shown that long-chain fatty acids of different classes act as signals to repress SPI1-encoded virulence through their action on the central transcriptional regulator HilD.^17^ ^,^ ^18^ ^,^ ^29^ The signals include common unsaturated fatty acids as well as DSFs, a rare class of *cis*-2-unsaturated long-chain fatty acids employed in quorum sensing.^43-45^ We sought to determine which class of long-chain fatty acid plays an important role in regulating expression of SPI1-encoded virulence factors in the gut, utilizing HilD mutants that are selectively responsive to specific classes of these molecules. Using an *in vivo* competition model (schematic Figure 1A), we first competed the *hilD* ^N44A^ mutant,^19^ which is unresponsive to all fatty acid classes, with the wild type in 129X1/SvJ mice, which can be infected and stably colonized with *Salmonella.* ^46^ We anticipated that *hilD* ^N44A^ would induce the uncontrolled expression of SPI1 genes and thus would suffer a fitness defect in the gut when compared to the wild type. We found that *hilD* ^N44A^ outcompeted the wild type at day 3 post-infection, but that the reverse was true from day 5 to day 14 of infection, with the wildtype significantly outcompeting this mutant on day 10 (Figure 1B).^47^ Next, to determine the specific class of fatty acid that exerts this control, we competed the wild type with a *hilD* ^Q290A^ mutant,^19^ which is unresponsive only to fatty acids that lack the *cis*-2-unsaturation. We found no significant difference in the proliferation of *hilD* ^Q290A^ compared to the wild type throughout the course of infection, suggesting that *cis*-2-unsaturated fatty acids play the major role in repressing SPI1-encoded virulence factors in the gut (Figure 1C). To ensure that the effects of *hilD* ^N44A^ were not simply due to inadequate HilD production or function in the gut, we additionally compared survival of the wild type to that of a Δ*hilD* null mutant strain (Figure 1D **)**. We found no difference between the two, indicating that the phenotype of *hilD* ^N44A^ could not be attributed to insufficient HilD activity. We additionally quantified inflammation levels in mice infected with the wild type in combination with the *hilD* ^N44A^, *hilD* ^Q290A^, or Δ*hilD* strain, by measuring fecal lipocalin concentration (Figure 1E). The *hilD* ^N44A^/wild type combination induced significantly higher lipocalin levels on days 3–5 post-infection compared to combinations including *hilD* ^Q290A^ or Δ*hilD* strains. These differences did not exist at later time points, as inflammation increased for all combinations of strains. The delayed inflammatory response generated by the combination of the wild type and the Δ*hilD* strain, in which only the wild type can induce inflammation, thus demonstrates that *hilD* ^N44A^ can aberrantly hasten the host response to *Salmonella*.

These results imply that fatty acid signals play an important role in repressing the HilD-regulated virulence factors to ameliorate the associated fitness cost, and thus that the *hilD* ^N44A^ mutant survives poorly due to its uncontrolled expression of virulence determinants. To test this directly, we next competed the wild type strain with a *hilD* ^N44A^ mutant in which the remaining SPI1 had been removed (Figure 1F). Here we found the opposite result: by 5 d post-infection, the *hilD* ^N44A^lacking SPI1 outcompeted the wild type, as would be expected if this strain were relieved of the metabolic burden imposed by unregulated production of virulence determinants. This strain, however, differs from the wild type in two respects: its poor expression of SPI1 genes, and its functional inability to invade intestinal epithelial cells, failing to induce inflammation. To determine which of these affected bacterial survival in the gut, we next compared the wild type to *hilD* ^N44A^ strain also lacking *sipD*, which encodes an essential translocon component (Figure 1G). This strain therefore fails to invade cells but is otherwise fully capable of expressing SPI1 genes. By day 9 post-infection, we recovered lesser *hilD* ^N44A^, Δ*sipD* strain than the wild type, indicating that the production of SPI1 determinants, rather than their effects within the host, lead to the fitness defect. As a final control, we compared a ΔSPI1 strain to an isogenic *hilD* ^N44A^, ΔSPI1 strain, finding, as expected, no fitness difference between these strains, as neither is able to produce virulence determinants (Figure 1H). Overall, these data indicate that fatty acid signals, likely of the DSF class, can play an important role in repressing SPI1-encoded virulence factors, promoting proliferation of *Salmonella* in the gut.

### *S. maltophilia* enhances murine colonization of *Salmonella*

**Figure 2.**
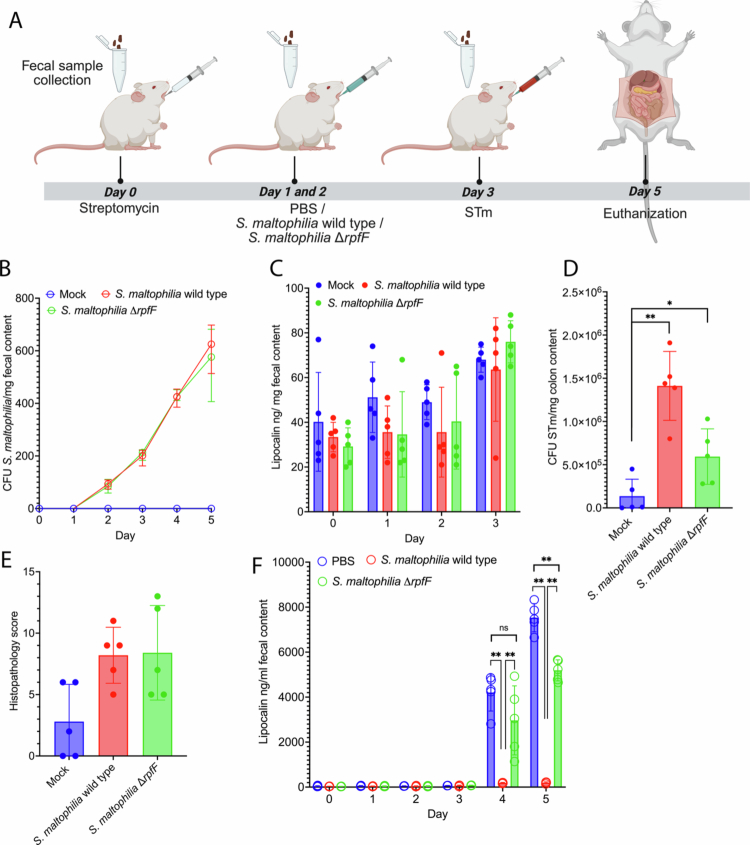
*S. maltophilia* enhances murine colonization of *Salmonella*. (a). Schematic diagram showing the events of *Salmonella* (STm) infection of mice pre-colonized with *S. maltophilia*, made in BioRender. Groups of 129X1/SvJ mice (*n* = 5) were orally gavaged with streptomycin on day 0 and inoculated with *S. maltophilia* wild type or Δ*rpfF* strains on days 1 and 2. Oral STm infection was performed on day 3. Mice were euthanized on day 5 to quantify STm colonization. Fecal samples collected throughout the experiment were plated on VIA plates to quantify *S. maltophilia.* (b). Quantification of *S. maltophilia* in fecal contents. Fecal pellets collected every day of the experiment were weighed, dissolved in PBS and spread on VIA plates to enumerate *S. maltophilia.* PBS was used as control (Mock). Line graph shows mean ± SD of bacterial numbers per mg fecal content. (c). Quantification of inflammation due to mock (PBS) or *S. maltophilia* wild type or Δ*rpfF* treatment by fecal lipocalin ELISA. Bars show mean ± SD of five biological replicates. (d). Quantification of STm infection in mice pre-inoculated with PBS or pre-colonized with *S. maltophilia* wild type or Δ*rpfF*. Colon contents collected from all groups on day 5 were weighed and spread on LB agar plates with kanamycin to determine the STm numbers. Bars show mean ± SD of five biological replicates. Differences between indicated groups were calculated by Mann–Whitney test. **p < 0.01; *p < 0.05, ns is not significant. (e). Histopathology scoring of intestinal damage due to STm infection of mice pre-inoculated with PBS or pre-colonized with *S. maltophilia* wild type or Δ*rpfF*. On day 5, after euthanization, sections of ileum, cecum and colon were sampled for histopathology. Damage to the cecum was scored in a blind manner. Bars show mean ± SD of five biological replicates. (f). Quantification of colonic inflammation in mice pre-inoculated with PBS or pre-colonized with *S. maltophilia* wild type or Δ*rpfF* after *Salmonella* infection by fecal lipocalin ELISA. Lipocalin levels from day 0 are shown as bars (mean ± SD of five biological replicates). **p < 0.01; ns is not significant.

As these results suggest that *Salmonella* utilizes *cis*-2 fatty acids as signals for murine colonization, we sought to identify the organisms of the native microbiota that can produce such signals. The signature *cis*-2 unsaturation that characterizes DSFs is catalyzed by a dehydratase/thioesterase encoded by *rpfF.* ^36^ We thus investigated which microbial species known to inhabit the gut carry *rpfF*. *S. maltophilia* harbors *rpfF*, produces DSFs^48^ ^,^ ^49^ and has been shown to be a prominent resident of human colonic crypts in one study,^41^ a potential commensal in additional studies,^38-40^ ^,^ ^50^ but due to its paucity in analyses of human feces, it is not yet recognized as a universal member of the gut microbiota. We thus tested the ability of *S. maltophilia* to facilitate *Salmonella* colonization and the importance of its DSFs to this effect (schematic Figure 2A). We used the same strain of mice, 129X1/SvJ, from the same vendor throughout these studies as they showed presence of *S. maltophilia* as part of their native microbiota (Figure S1). Groups of 129X1/SvJ mice were orally inoculated with *S. maltophilia* wild type or Δ*rpfF* after streptomycin treatment to reduce existing microbiota and facilitate *S. maltophilia* establishment and then orally infected with *Salmonella*. Streptomycin treatment is a widely used and accepted model for reduction of the native microbiota to facilitate enteric infections. This model differs from the natural colonization 129X1/SvJ model used in Figure 1. The streptomycin pretreatment is required here to establish consistent and reproducible *S. maltophilia* colonization, whereas the natural-colonization model in Figure 1 isolates the long-term fitness cost of SPI-1 expression in an intact microbiota; the two models are thus complementary, addressing distinct biological questions.^51-54^ We found that both *S. maltophilia* strains successfully colonized the colon at similar rates of ~2 × 10^2^ CFU/milligram of feces (Figure 2B). Additionally, *S. maltophilia* did not itself cause detectable inflammation of the colon, as measured by fecal lipocalin levels (Figure 2C). Wild type *S. maltophilia*, however, promoted *Salmonella* colonization in high numbers, ~10-fold greater than for the control mice that received no *S. maltophilia* (Figure 2D). The *S. maltophilia* Δ*rpfF* mutant supported more modest levels of *Salmonella* colonization (~4-fold), while both strains promoted mucosal damage and acute inflammation induced by *Salmonella* (Figure 2E). These two strains, however, varied greatly in their effects on the gut inflammation that accompanies *Salmonella* infection. The presence of wild type *S. maltophilia* in the gut greatly reduced the level of colonic lipocalin induced by *Salmonella* infection, by ~22-fold and ~39-fold on days 4 and 5 of the assay, respectively (Figure 2F). This repression was lacking in mice with the Δ*rpfF* mutant, in which lipocalin levels did not differ from those of the uninoculated control mice. These results thus not just show that *S. maltophilia* in the murine colon fosters colonization by *Salmonella*, but that this organism can also quell an important host response to *Salmonella* infection in an *rpfF*-dependent manner.

### *S. maltophilia* produces c2-HDA under oxidative stress

**Figure 3.**
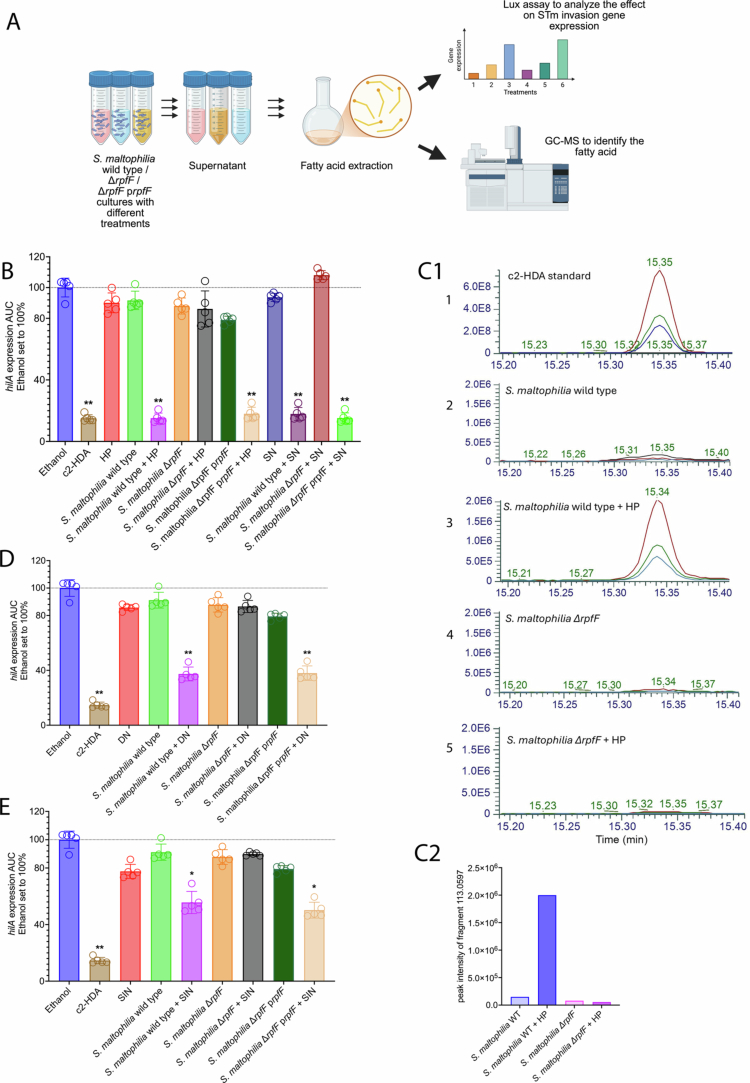
*S. maltophilia* produces c2-HDA under oxidative stress. (a). Schematic diagram summarizing the process of fatty acid extraction and identification, made in BioRender. *S. maltophilia* wild type or Δ*rpfF* or Δ*rpfF* p*rpfF* cultures were grown in MGAM medium at 30 °C for 12 h with hydrogen peroxide (HP) or spermine NONOate (SN). Fatty acids extracted from culture supernatants were added to growing STm cultures and the effect on invasion gene expression was analyzed by lux assay. Selected extracts were derivatized and analyzed by GC–MS to identify the fatty acids. (b). Effect of the extracted fatty acids on STm invasion gene expression. Extracted fatty acids were added to growing STm cultures having a *hilA-luxCDABE* transcriptional reporter fusion. c2-HDA was used as a control at 2 µM concentration. Luminescence was normalized to the bacterial culture density. The area under curve (AUC) for each treatment was calculated. The AUC for the solvent control ethanol was set to 100%, and others were normalized accordingly. Bars show the normalized AUC ± SD (*n* = 5). Differences between ethanol and other treatments were calculated by Mann–Whitney test. **p < 0.01. C1-2. Extracted ion chromatograms (EICs, C1) of fatty acid samples, highlighting a key DSF peak at a retention time of ~15.35 min. Overlayed EICs are shown for *m/z* 113.0591 (red), 81.0331 (green), and 171.1370 (blue) ± 5 ppm. 1. c2-HDA standard; 2–5. fatty acids extracted from culture supernatants of: *S. maltophilia* wild type grown with (2) and without HP (3), and Δ*rpfF* mutant grown with (4) and without HP (5). In C2 the quantification of 113.0591 in different samples is shown as bar graph. (d and e). Effect of the extracted fatty acids on STm invasion gene expression. *S. maltophilia* wild type or Δ*rpfF* or Δ*rpfF* p*rpfF* cultures were grown with (d) DETA-NONOate (DN) or (e) SIN-1 (SIN) at 30 °C. Fatty acids extracted from culture supernatants were added to growing STm cultures having a *hilA-luxCDABE* transcriptional reporter fusion. c2-HDA was used as a control at 2 µM concentration. Luminescence was normalized to the bacterial culture density. The area under curve (AUC) for each treatment was calculated. The AUC for the solvent control ethanol was set to 100%, and others were normalized accordingly. Bars show the normalized AUC ± SD (*n* = 5). Differences between ethanol and other treatments were calculated by Mann–Whitney test. *p < 0.05 and **p < 0.01.

As *S. maltophilia* can alter the inflammatory behavior of *Salmonella*, likely through the production of a chemical signal, we reasoned that the converse might also be true: that the inflammatory environment created by *Salmonella* might elicit *S. maltophilia* to produce this same signal, thus creating the possibility of a signaling feedback loop. To initially test this, we grew *S. maltophilia* in differing concentrations of hydrogen peroxide (HP), a potent reactive oxygen species (ROS), extracted the fatty acids from the culture supernatant and added them to growing *Salmonella* cultures to analyze invasion-gene expression using a *hilA-luxCDABE* reporter fusion (schematic Figure 3A). We found that fatty acids extracted from *S. maltophilia* grown in laboratory medium had no effect on this reporter. Extracts of *S. maltophilia* grown with the addition of 30 μM HP, however, greatly reduced *Salmonella hilA* expression, by ~7-fold (Figure 3B). GC–MS analysis of the fatty acid extract confirmed in these extracts the presence of c2-HDA (Figures 2 and 3), the most potent invasion gene repressor of *Salmonella.* ^19^ ^,^ ^23^ ^,^ ^37^ Fatty acid extracts from *S. maltophilia* Δ*rpfF*grown under the same conditions, with 30 μM HP or without, failed to substantially reduce *Salmonella hilA* expression, however the phenotype was reversed to wild type levels (~6 fold) by complementation of *rpfF* in a low copy plasmid (Δ*rpfF* p*rpfF*) (Figure 3B). Extracts from the Δ*rpfF* mutants also lacked the characteristic peak of c2-HDA by GC–MS, showing that *rpfF* alone is responsible for c2-HDA production (Figures 2 and 3). c2-HDA is the predominant DSF-family molecule we detected in our *S. maltophilia* extracts; the canonical DSF *cis*-11-methyl-2-dodecenoic acid (c11-Me-DDA), which is also synthesized by RpfF, was not detected in our extractions. Further, analysis of *hilA* expression showed that this chemical can moderately repress *Salmonella* virulence but is significantly less potent than c2-HDA (Figure S2).

To validate the link between oxidative stress and c2-HDA production, we exposed *S. maltophilia* instead to spermine NONOate (SN), a compound known to generate reactive nitrogen species (RNS) or nitrosative stress,^55^ ^,^ ^56^ and again observed robust reduction (by ~6 fold) in *Salmonella hilA* expression (Figure 3B). To further distinguish which RNS: nitric oxide (NO) or peroxynitrite (ONOO^−^), is the primary inducer of c2-HDA production by *S. maltophilia*, cultures were treated separately with the selective NO donor DETA-NONOate (diethylenetriamine NONOate, hereafter DN) or with the peroxynitrite donor SIN-1 (3-morpholinosydnonimine hydrochloride, hereafter SIN). DN slowly liberates NO (half-life ~20 h) under conditions that minimize peroxynitrite formation, whereas SIN concurrently generates NO and superoxide (O₂^−^), which combine spontaneously to yield ONOO^−^. We observed a ~3 fold reduction in *Salmonella hilA* expression upon treatment with fatty acids extracted from *S. maltophilia* cultures treated with DN (Figure 3D). Treatment with SIN, although statistically significant, led to only ~2 fold repression, highlighting that NO is the predominant RNS than peroxynitrite for c2-HDA induction (Figure 3E). These results collectively confirm that oxidative stress is a potent trigger for c2-HDA biosynthesis in *S. maltophilia in vitro*, providing a potential link between inflammation and microbial signaling in the colon.

### Inflammation generated by macrophages can drive c2-HDA production by *S. maltophilia*

**Figure 4.**
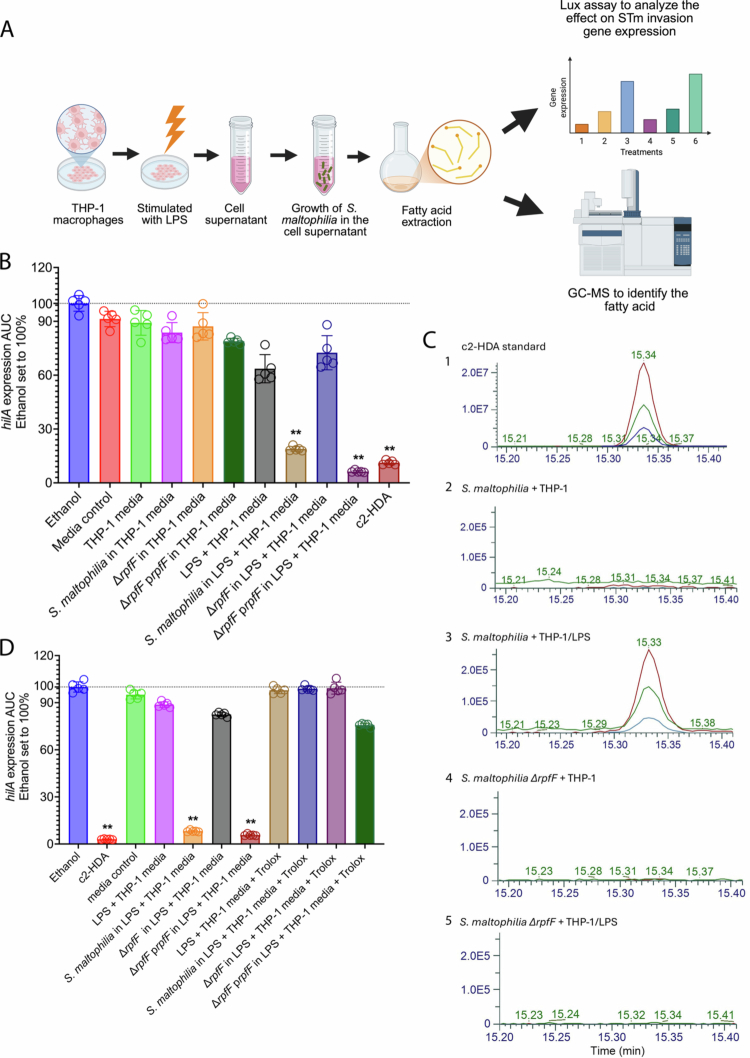
Inflammation generated by macrophages can drive c2-HDA production by *S. maltophilia*. (a). Schematic diagram summarizing the experimental setup, made in BioRender. THP-1 macrophages were stimulated with LPS. Cell supernatant was collected and *S. maltophilia* wild type or Δ*rpfF* or Δ*rpfF* p*rpfF* were grown in it at 37 °C. Bacteria were pelleted and fatty acids were extracted from the culture supernatant. These were added to growing STm cultures and the effect on invasion gene expression was analyzed by lux assay. Selected extracts were derivatized and analyzed by GC–MS to identify the fatty acids. (b). Effect of the extracted fatty acids on STm invasion gene expression. *S. maltophilia* wild type or Δ*rpfF* or Δ*rpfF* p*rpfF* were grown at 37 °C in the cell supernatant of THP-1 macrophages stimulated or not with LPS. Extracted fatty acids from these *S. maltophilia* cultures were added to growing STm cultures having a *hilA-luxCDABE* transcriptional reporter fusion. c2-HDA was used as a control at 2 µM concentration. Luminescence was normalized to the bacterial culture density. The area under curve (AUC) for each treatment was calculated. The AUC for the solvent control ethanol was set to 100%, and others were normalized accordingly. Bars show the normalized AUC ± SD (*n* = 5). Differences between ethanol and other treatments were calculated by Mann–Whitney test. **p < 0.01. (c). Extracted ion chromatograms (EICs) of fatty acid samples, highlighting a key DSF peak at a retention time of ~15.35 min. Overlayed EICs are shown for *m/z* 113.0591 (red), 81.0331 (green), and 171.1370 (blue)±5 ppm. 1. c2-HDA standard; 2-5. fatty acids extracted from culture supernatants of: *S. maltophilia* wild type grown in cell supernatant of unstimulated THP-1 macrophages (2) or stimulated with LPS (3); Δ*rpfF* mutant grown in cell supernatant of unstimulated THP-1 macrophages (4) or stimulated with LPS (5). (d). Effect of addition of the antioxidant Trolox. Cell supernatants of THP-1 macrophages stimulated with LPS with or without Trolox treatment were collected. *S. maltophilia* wild type or Δ*rpfF* or Δ*rpfF* p*rpfF* were grown at 37 °C in these cell supernatants and fatty acids were extracted. These were added to growing STm cultures having a *hilA-luxCDABE* transcriptional reporter fusion. c2-HDA was used as a control at 2 µM concentration. Luminescence was normalized to the bacterial culture density. The area under curve (AUC) for each treatment was calculated. The AUC for the solvent control ethanol was set to 100%, and others were normalized accordingly. Bars show the normalized AUC ± SD (*n* = 5). Differences between ethanol and other treatments were calculated by Mann–Whitney test. **p < 0.01.

Our results demonstrate that under laboratory conditions *S. maltophilia* produces c2-HDA upon exposure to oxidative stress. As similar stress is produced *in vivo* by macrophages upon exposure to invading bacteria,^57^ we hypothesized that the inflammation produced by macrophages upon encountering *Salmonella* can act as a signal for *S. maltophilia* to produce c2-HDA. To test this, we used THP-1 monocytes differentiated into macrophages using PMA (hereafter THP-1 macrophages) and stimulated with LPS.^58^ Production of ROS and RNS in the culture supernatant, was confirmed using the Amplex Red and Greiss reagent assay respectively (Figures S3 and S4). We grew *S. maltophilia* in culture supernatants of these stimulated cells, extracted fatty acids, and added the extracts to *Salmonella* cultures to analyze *hilA* expression and fatty acid production (schematic Figure 4A). We found that fatty acid extracts from wild type and complemented *S. maltophilia* significantly reduced *Salmonella* invasion gene expression (~5 fold and ~16 fold respectively, Figure 4B), and that c2-HDA was present (Figures 4C and S7A). In contrast, c2-HDA was absent in the fatty acid extracts from the Δ*rpfF* mutant strain grown in identical conditions and hence failed to repress *hilA* expression. In addition, wild type and complemented *S. maltophilia* grown in culture supernatants from unstimulated THP-1 macrophages failed to repress *hilA* expression. These results show the specificity of c2-HDA production by *S. maltophilia* under the inflammatory conditions generated *in vitro* by LPS.

We next sought to determine whether the ROS/RNS in the culture supernatants of the LPS-stimulated THP-1 macrophages were the primary triggers for c2-HDA production by *S. maltophilia*. We thus quenched the oxidative species present in these supernatants using the antioxidant Trolox, a water-soluble *α*-tocopherol analog that functions as a broad-spectrum scavenger of both ROS (hydroxyl radical, peroxyl radical, lipid peroxides) and RNS (NO, peroxynitrite) through hydrogen atom transfer.^59-62^ Reduction in ROS and RNS quantities in the culture supernatant were confirmed by Amplex Red and Greiss reagent assay respectively (Figures S3 and S4). The addition of antioxidants severely impaired the ability of fatty acids extracted from wild type and complemented *S. maltophilia* to reduce *hilA* expression; samples without Trolox treatment showed ~12-fold and ~18 fold repression respectively, whereas treated samples showed no significant difference compared to the solvent control. In fact, addition of Trolox restored *hilA* expression to the level found for the mutant Δ*rpfF* strain (Figure 4D). Together, these results provide evidence that *S. maltophilia* can respond to the oxidative signals generated by THP-1 macrophages, producing c2-HDA specifically in response to immune-mediated stress.

### Inflammation induced by *Salmonella* exposure induces c2-HDA production

**Figure 5.**
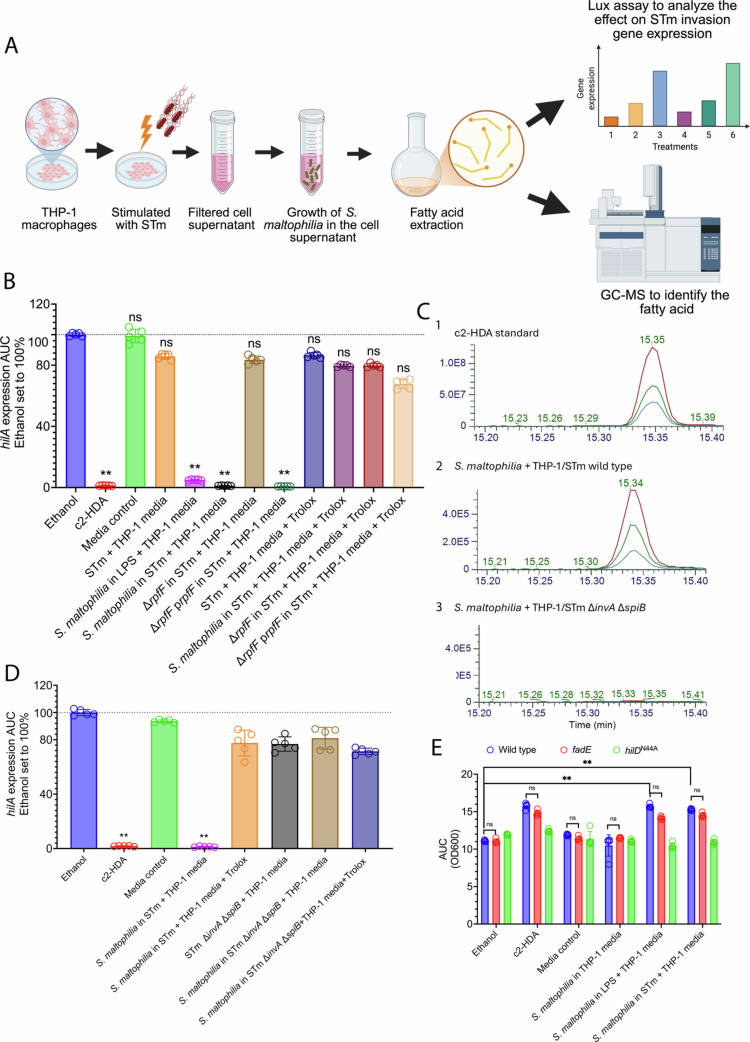
Inflammation induced by *Salmonella* exposure induces c2-HDA production. (a). Schematic diagram summarizing the experimental setup, made in BioRender. THP-1 macrophages were stimulated with STm wild type or Δ*invA* Δ*spiB*. Cell supernatant was collected and filtered to remove bacteria. *S. maltophilia* wild type or Δ*rpfF* or Δ*rpfF* p*rpfF* were grown in the filtered cell supernatant at 37 °C. Bacteria were pelleted and fatty acids were extracted from the culture supernatant. These were added to growing STm cultures and the effect on invasion gene expression was analyzed by lux assay. Selected extracts were derivatized and analyzed by GC–MS to identify the fatty acids. (b). Effect of THP-1 macrophage stimulation with STm. *S. maltophilia* wild type or Δ*rpfF* or Δ*rpfF* p*rpfF* were grown in the filtered cell supernatant at 37 °C of THP-1 macrophages stimulated with LPS or STm with or without Trolox treatment. Extracted fatty acids from these *S. maltophilia* cultures were added to growing STm cultures having *hilA-luxCDABE* transcriptional reporter fusion. c2-HDA was used as a control at 2 µM concentration. Luminescence was normalized to the bacterial culture density. The area under curve (AUC) for each treatment was calculated. The AUC for the solvent control ethanol was set to 100%, and others were normalized accordingly. Bars show the normalized AUC ± SD (*n* = 5). Differences between ethanol and other treatments were calculated by Mann–Whitney test. **p < 0.01; ns is not significant. (c). Extracted ion chromatograms (EICs) of fatty acid samples, highlighting a key DSF peak at a retention time of ~15.35 min. Overlayed EICs are shown for *m/z* 113.0591 (red), 81.0331 (green), and 171.1370 (blue) ± 5 ppm. 1. c2-HDA standard; 2–3. fatty acids extracted from culture supernatants of *S. maltophilia* grown in cell supernatant of THP-1 macrophages stimulated with STm wild type (2) or Δ*invA* Δ*spiB* (3). (d). Effect of THP-1 macrophage stimulation with STm wild type or Δ*invA* Δ*spiB*. Cell supernatants of THP-1 macrophages stimulated with STm wild type or Δ*invA* Δ*spiB* with or without Trolox treatment were collected and filtered. *S. maltophilia* were grown in these cell supernatants at 37 °C and fatty acids were extracted. These were added to growing STm cultures having a *hilA-luxCDABE* transcriptional reporter fusion. c2-HDA was used as a control at 2 µM concentration. Luminescence was normalized to the bacterial culture density. The area under curve (AUC) for each treatment was calculated. The AUC for the solvent control ethanol was set to 100%, and others were normalized accordingly. Bars show the normalized AUC ± SD (*n* = 5). Statistical differences between ethanol and indicated datasets were calculated by Mann–Whitney test. **p < 0.01. (e). Effect of fatty acids on growth rate of STm. *S. maltophilia* were grown in the filtered cell supernatant of THP-1 macrophages at 37 °C stimulated with LPS or STm. Extracted fatty acids from these *S. maltophilia* cultures were added to growing STm cultures. c2-HDA was used as a control at 2 µM concentration. OD600 was monitored for 24 h and measurements were recorded every half-hour. The area under curve (AUC) for each treatment was calculated. Bars show AUC ± SD (*n* = 5). Differences between ethanol and other treatments or indicated groups were calculated by Mann–Whitney test. **p < 0.01; ns is not significant.

The results described above demonstrate that ROS/RNS generated by LPS-stimulated THP-1 macrophages can induce *S. maltophilia* to produce c2-HDA in culture. However, the intestinal environment presents a more complex context, where the interaction between the host and the pathogen is complicated by spatial, temporal, and ecological factors. We hypothesized that during *Salmonella* infection, the inflammatory response initiated by colonic macrophages triggers *S. maltophilia* in the colon to produce c2-HDA. This, in turn, is utilized by *Salmonella* to control its virulence and switch to proliferation. To test this hypothesis, we designed an *in vitro* model that more closely resembles the probable sequence of events in the intestine. THP-1 macrophages were stimulated with *Salmonella* and the resulting culture supernatants were used to grow *S. maltophilia*, followed by fatty acid extraction and testing of those extracts. THP-1 macrophages were left untreated as a non-inflammatory control (schematic Figure 5A). We found that the fatty acids extracted from wild type and complemented *S. maltophilia* cultures grown in the supernatants of *Salmonella*-infected THP-1 macrophages significantly repressed (~84 and ~134 fold respectively) *Salmonella* invasion expression (Figure 5B) and contained c2-HDA (Figures 5C and S7B). The repression levels were comparable to those obtained with wild type *S. maltophilia* grown in LPS-treated THP-1 macrophages. In contrast, treatment with Trolox ameliorated these effects, demonstrating that *Salmonella* mediated oxidative stress triggers *S. maltophilia* to produce c2-HDA. The type 3 secretion systems 1 and 2 (T3SS-1 and 2) in *Salmonella* are responsible for invasion into intestinal epithelial cells and survival within macrophages, respectively, and are known drivers of intestinal inflammation.^63^ ^,^ ^64^ To test whether this source of inflammation was required for the effects we observed, we exposed THP-1 macrophages to a *Salmonella* strain devoid of functional T3SS-1 and 2 (Δ*invA*, Δ*spiB*). We found that fatty acids extracted from *S. maltophilia* failed to repress *Salmonella hilA* expression and did not show the presence of c2-HDA (Figure 5C and D). These findings thus indicate that *Salmonella*-mediated inflammation is capable of inducing *S. maltophilia* to produce c2-HDA.

We next hypothesized that by suppressing its virulence cascade *Salmonella* may conserve energy for proliferation, an important function as the pathogen exits the host via feces. We therefore tested the growth rate of *Salmonella* in presence of extracts from *S. maltophilia* grown in culture supernatants of stimulated THP-1 macrophages. We found that fatty acids extracted from *S. maltophilia* cultures significantly increased *Salmonella* growth (Figure 5E). This surge might be attributed to fatty acids being used as sources of carbon via β-oxidation. Therefore, we used a mutant strain of *Salmonella* unable to use fatty acids as a carbon source (Δ*fadE*)^17^. Notably, we found that the growth rate of the Δ*fadE* mutant was comparable to that of wild type *Salmonella*, demonstrating that the increase in growth was unrelated to fatty-acid consumption (Figure 5E). We proposed instead that c2-HDA-mediated repression of the metabolically expensive SPI-1 program, which has been measured to slow the growth of SPI-1-expressing cells by approximately 25%,^6^ thus liberating cellular resources for proliferation. To test this model, we repeated the growth assay with *hilD* ^N44A^ strain which is unable to utilize c2-HDA for virulence repression. Consistent with this model we found that, unlike the wild type *Salmonella* strain, growth of the *hilD* ^N44A^ mutant was not enhanced in the presence of culture supernatants containing c2-HDA (Figure 5E). Together, these findings demonstrate that *Salmonella* infection of macrophages triggers a robust ROS/RNS inflammatory response, stimulating *S. maltophilia* to produce c2-HDA, which in turn is exploited by *Salmonella* to control its invasion and increase proliferation.

### The ability to recognize c2-HDA produced by *S. maltophilia* is important for *Salmonella* colonization

**Figure 6.**
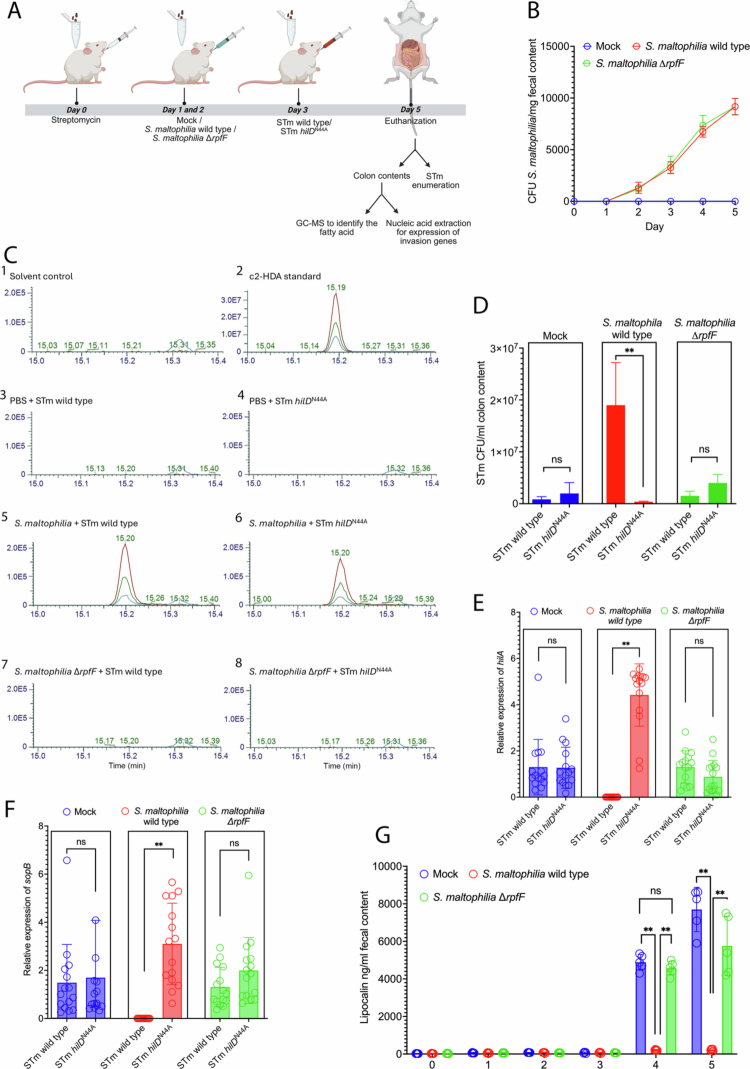
The ability to recognize c2-HDA produced by *S. maltophilia* is important for *Salmonella* colonization. (a). Schematic diagram showing infection with STm wild type or STm *hilD* ^N44A^ in mice pre-inoculated with PBS or pre-colonized with *S. maltophilia*. Groups of 129X1/SvJ mice (*n* = 5) were orally gavaged with streptomycin on day 0 and inoculated with *S. maltophilia* wild type or Δ*rpfF* strains on days 1 and 2. Oral STm infection was performed on day 3. Mice were euthanized on day 5 to quantify STm colonization. Fecal samples collected throughout the experiment were plated on VIA plates to quantify *S. maltophilia.* This diagram was created in BioRender. (b). Quantification of *S. maltophilia* in fecal contents. Fecal pellets collected every day of the experiment were weighed, dissolved in PBS and spread on VIA plates to enumerate *S. maltophilia.* PBS was used as control (Mock). Line graph shows mean ± SD of bacterial numbers per mg fecal content. (c). Extracted ion chromatograms (EICs) of fatty acid samples extracted from murine colon contents, highlighting a key DSF peak at a retention time of ~15.20 min. Overlayed EICs are shown for *m/z* 113.0591 (red), 81.0331 (green), and 171.1370 (blue) ±5 ppm. 1. Solvent control; 2. c2-HDA standard; 3–8. fatty acids extracted from colon contents of mice pre-inoculated with: PBS and infected with STm wild type (3) or *hilD* ^N44A^ (4); *S. maltophilia* wild type and infected with STm wild type (5) or *hilD* ^N44A^ (6); *S. maltophilia* Δ*rpfF* and infected with STm wild type (7) or *hilD* ^N44A^ (8). (d). Quantification of STm wild type or *hilD* ^N44A^ in each group of mice, pre-inoculated with PBS or pre-colonized with *S. maltophilia* wild type or Δ*rpfF*. On day 5, after euthanization, colon contents were weighed and spread on LB agar plates with kanamycin (for wild type) and chloramphenicol (for *hilD* ^N44A^) to determine the STm numbers and were normalized to per ml colon content. Bars show mean ± SD of five biological replicates. Difference between indicated groups were calculated by Mann–Whitney test. **p < 0.01; ns is not significant. (e and f). Relative gene expression of *hilA* (e) and *sopB* (f) in STm wild type or *hilD* ^N44A^ recovered from murine colon contents were measured by RT-qPCR. CT values were normalized to the mean of *gapA*. Data show mean ± SD of five biological replicates each having three technical replicates (total *n* = 15). Differences between the mock (PBS) and the *S. maltophilia* pre-inoculations were calculated by the Mann–Whitney test. **p < 0.01. ns is not significant. (g). Quantification of colonic inflammation in mock or *S. maltophilia* wild type or Δ*rpfF* pre-colonized mice after STm WT infection by fecal lipocalin ELISA. Bars show mean ± SD of five biological replicates. **p < 0.01; ns is not significant.

Our experimental findings indicate that *Salmonella* benefits from intestinal signals that suppress its virulence and promote proliferation in the large intestine. To determine whether *S. maltophilia* facilitates this transition by producing c2-HDA *in vivo*, we inoculated streptomycin-pretreated mice with either the wild type *S. maltophilia*, its Δ*rpfF* mutant (deficient in c2-HDA production), or a sham control. From five mice in each group (*n* = 10), colon contents were collected to evaluate c2-HDA production by GC–MS (Figure S5). The remaining mice in each group were then infected with either *Salmonella* wild type or *hilD* ^N44A^, which is unresponsive to fatty acid signals. After 48 h, mice were euthanized, and colon contents were collected. Bacterial loads of *Salmonella* wild type and *hilD* ^N44A^ were determined by selective antibiotic plating, while luminal contents were extracted for detection of c2-HDA by GC–MS, and invasion gene expression was assessed by RT-qPCR of the *hilA* and *sopB* transcripts (schematic Figure 6A). Although both *S. maltophilia* strains colonized at similar levels (Figure 6B), c2-HDA was detectable only in the colons of mice colonized with the wild type *S. maltophilia* strain (Figures 6C and S5). In these mice, wild type *Salmonella* exhibited significantly higher colonization (~65 fold greater than *hilD* ^N44A^; Figure 6D). This competitive advantage was lost in the presence of the *S. maltophilia* Δ*rpfF* mutant, where both the wild type and *hilD* ^N44A^ *Salmonella* were recovered at similar levels (~0.3 fold greater than *hilD* ^N44A^), indicating that the ability to utilize c2-HDA produced by *S. maltophilia* confers a fitness benefit *in vivo*. Notably, in contrast to our findings *in vitro*, *S. maltophilia* produced c2-HDA *in vivo* independent of *Salmonella*-induced inflammation (Figure S5), suggesting additional means of DSF induction in the gut environment.

To further validate the regulatory role of c2-HDA, we quantified expression of the invasion genes *hilA* and *sopB* in the luminal *Salmonella* across all groups. In sham-treated and Δ*rpfF*-colonized mice, invasion gene expression remained similarly elevated in both the wild type and *hilD* ^N44A^ strains (Figure 6E and F). However, in the presence of c2-HDA-producing *S. maltophilia*, only the wild type *Salmonella* exhibited a discernable reduction in *hilA* and *sopB* expression, consistent with its ability to respond to this signal. These findings underscore the functional relevance of c2-HDA sensing for downregulating energetically costly virulence *in vivo*.

Finally, we assessed inflammation by measuring fecal lipocalin-2 levels. In mice colonized with the wild type *S. maltophilia*, lipocalin levels were significantly reduced relative to mice receiving no *S. maltophilia* or the Δ*rpfF* mutant (Figure 6G), indicating that suppression of virulence via c2-HDA also mitigates host inflammatory responses. Together, these results demonstrate that *Salmonella* can respond to microbiota-derived signals such as c2-HDA to strategically modulate its virulence program, enhance colonization, and exploit inflammation-augmented microbial signaling to optimize its fitness within the host gut.

## Discussion

*Salmonella* has evolved intricate mechanisms to exploit environmental molecules in the gut as signals to refine its virulence programs. Among these, the colonic fatty acid c2-HDA has emerged as a key modulator, previously shown to potently repress invasion gene expression and attenuate *Salmonella* virulence. Here we demonstrate that *Salmonella* is capable of sensing and responding to signals generated by the microbiota in response to its invasion to facilitate its persistence. Notably, we identify *S. maltophilia* as the microbial contributor to this process, as its presence in the murine gut significantly enhances *Salmonella* infection. Mechanistically, we show that *S. maltophilia* enhances the production of c2-HDA in response to inflammation-induced oxidative stress, a response that depends on *Salmonella's* T3SS-1 and T3SS-2. Furthermore, the ability of *Salmonella* to utilize these fatty acids is essential to maximize colonization of the inflamed gut in murine models. Together, these findings reveal a tightly regulated feedback loop, engaging the pathogen, the host, and the microbiota, wherein *Salmonella*-induced inflammation can amplify the production of a microbiota-derived metabolite that the pathogen subsequently detects to refine its colonization program. This work suggests a novel signaling axis by which a pathogen may co-opt a host-microbiota signaling network to support its transmission fitness.

Colonization resistance provided by the native gut microbiota is one of the fundamental means of protection against pathogen invasion. This protective effect is conferred by several mechanisms, including competition for nutrients and mucosal niches or attachment sites, as well as by strengthening the immune system, which enhances host innate immunity.^65^ ^,^ ^66^ However, this interaction is not unanimously antagonistic to pathogens. Certain commensal species produce metabolic byproducts that are exploited by the invading bacteria for their benefit. For example, the commensal *Bacteroides thetaiotaomicron* ferments carbohydrates to produce succinate that is exploited by the pathogen *Clostridium difficile* as a carbon source to support its expansion.^67^ Furthermore, *B. thetaiotaomicron* contributes to the production of mucosal monosaccharides fucose and sialic acid, which are utilized by pathogens *Clostridium difficile* and *Salmonella* to fuel their proliferation.^68^ These findings challenge the wide-spread notion of commensals as fundamentally protective, revealing instead a more nuanced landscape in which some native commensals may act as unwitting co-conspirators of pathogen colonization.

Inflammation is widely recognized as the primary component of the immune response, orchestrated to detect, contain, and eliminate invading pathogens, and thus is known to be a protective mechanism.^69^ However, several studies have shown that pathogens can drive as well as manipulate inflammation for their proliferation. In particular, *Shigella flexneri* secretes into host epithelial cells a specialized set of effector proteins that directly manipulate innate immune responses to promote bacterial colonization and survival.^70^ ^,^ ^71^ In addition, *Citrobacter rodentium* promotes hyperplasia in murine colonic crypts through its T3SS for aerobic expansion.^72^ Similarly, nitrate produced during inflammation is exploited by pathogenic *E. coli and Salmonella* as a respiratory electron acceptor, providing a selective fitness advantage to these pathogens.^13^ ^,^ ^73^ ^,^ ^74^ For *Salmonella*, the ROS generated as an inflammatory response to neutralize the pathogen is utilized as tetrathionate and exploited as a terminal electron acceptor by the pathogen for proliferation.^75^ ^,^ ^76^ Our findings support this concept and extend it to involve a member of the gut microbiota. Inflammation generated via *Salmonella* T3SS-1 and T3SS-2 may augment the production of c2-HDA from *S. maltophilia*, a fatty acid that *Salmonella* can employ to inhibit further expression of virulence and shift instead to replication. This is an important strategy that ensures higher proliferation of *Salmonella* by removing the energetic burden of producing virulence factors. Together, these findings suggest that gut inflammation functions in ways beyond its traditional role as a pathogen-neutralizing process, as a signaling component capable of shaping pathogen-microbiota interactions and inadvertently promoting the success of well-adapted pathogens.

Some caveats temper the interpretation of these findings. First, our *in vitro* experiments using THP-1 macrophages provide proof-of-principle mechanistic evidence that oxidative and nitrosative signals from activated phagocytes are sufficient to trigger c2-HDA production, but they do not exclude additional contributions from other intestinal cell types. Second, *S. maltophilia* may receive ROS/RNS from several sources in the inflamed gut, in addition to those generated by the *Salmonella*-macrophage interaction shown in his study, including neutrophils (via NOX2 and myeloperoxidase-derived hypohalous acids), epithelial cells (via NOX1 and DUOX2) and inflammatory monocytes. Third, at the 4 h post-infection time point used in our macrophage assays, T3SS-1 is the dominant driver of macrophage activation, with T3SS-2 contributing only modestly because its intracellular replication phenotype typically manifests at later time points. Our Δ*invA* Δ*spiB* double mutant therefore eliminates both systems, but the loss-of-induction phenotype primarily reflects loss of T3SS-1. Within macrophages, T3SS-1-translocated effectors (SipB) and flagellin/needle subunits activate the NLRC4/NAIP inflammasome,^77-79^ driving caspase-1-dependent IL-1β/IL-18 release, NADPH oxidase (NOX2) assembly and iNOS induction, thereby generating the ROS/RNS detected in our supernatants. Fourth, the streptomycin pretreatment used in our *in vivo* experiments itself induces low-grade intestinal inflammation and dysbiosis,^51^ ^,^ ^80^ which can serve as an independent stimulus for c2-HDA production (Figure S5); this likely contributes to the *Salmonella*-independent c2-HDA production we observe *in vivo* and makes it difficult to isolate a specific *Salmonella*-driven contribution. Fifth, *S. maltophilia* is itself a well-documented inducer of macrophage activation as both an extracellular and intracellular organism,^81-83^ and in a native co-colonization setting may stimulate low-grade inflammation on its own that primes c2-HDA production in a feed-forward manner; *Salmonella* co-infection likely amplifies this baseline inflammation further, enhancing c2-HDA induction. Our reductionist *in vitro* design was necessary to isolate the *Salmonella* to macrophage to *S. maltophilia* axis, but the *in vivo* situation likely reflects a more complex, multi-source inflammatory environment.

Our findings underscore as well the adaptive nature of *S. maltophilia* metabolism, which is likely exploited by *Salmonella* for its virulence. It is plausible that *Salmonella'*s ability to respond to microbiota-derived c2-HDA is consistent with, but is not direct evidence of, a co-evolutionary adaptation; our study does not directly test evolutionary hypotheses. Published works^84^ ^,^ ^85^ show that DSF/c2-HDA signaling is widespread and functionally significant in *S. maltophilia*, including regulation of virulence and biofilm formation via *rpf*-mediated quorum sensing. This, combined with the ecological presence of *S. maltophilia* in the mammalian gut^40^ ^,^ ^41^ and its impact on host inflammation (Figure 6), supports a model where *Salmonella* may have evolved to sense such inter-bacterial cues to optimize its colonization and virulence strategy. Thus, our findings are consistent with an adaptive, possibly co-evolutionary interaction between host-associated bacteria and enteric pathogens.

## Star methods

### Bacterial strains, cells, and growth conditions

All experiments were performed using either *S. maltophilia* E77, *Salmonella enterica* serovar Typhimurium 14028s or their isogenic mutants, listed in Table 1. *S. maltophilia* E77 wild type and Δ*rpfF* were kindly provided by Dr. Daniel Yero.^86^ pRAB101e derived plasmid vector (A086; made by Dr. Artur Pinski) used for construction of the complemented *S. maltophilia* strain Δ*rpfF* p*rpfF* was donated by Dr. Anthony Hay. *Salmonella* strains were maintained on LB agar plates and grown in LB broth at 37 °C overnight with aeration. *S. maltophilia* cultures were maintained on LB plates and grown in Modified Gifu Anaerobic Medium (MGAM, from HiMedia) at 30 °C overnight. Selection of *S. maltophilia* from gut contents was done on *Stenotrophomonas* Selective Agar Base plates (HiMedia) supplemented with 5 µg/ml vancomycin, 32 µg/ml imipenem, and 2.5 µg/ml amphotericin (henceforth referred to as VIA agar plates).

**Table 1.** Strains used in this study.

Strain	Description	Source
CA32	*Salmonella* Typhimurium 14028s wild type	ATCC
CA569	*E.coli* S17.1 λpir	P. Orndorff
CA2946	Wild type, *philA-luxCDABE*	[15]
CA6033	Δ*invA:cam* Δ*spiB:kan*	This study
CA4622	Δ*fadE*, *philA-luxCDABE*	[17]
CA5731	*malXY:kan phoN:BFP* P*sicA → GFP*	This study
CA6079	*hilD* ^N44A^ malXY:cam phoN:BFP PsicA → GFP	This study
CA5255	*hilD* ^N44A^ malXY:cam	This study
CA5268	*hilD* ^Q290A^ malXY:cam	This study
CA5770	*S. maltophilia* E77 wild type	D. Yero [86]
CA5771	*S. maltophilia* E77 Δ*rpfF*	D. Yero [86]
RC100	*S. maltophilia* E77 Δ*rpfF* p*rpfF*	This study
CA6203	ΔSPI1 *hilD* ^N44A^ *malXY:cam*	This study
CA6204	Δ*sipD hilD* ^N44A^ *malXY:cam*	This study
CA4434	*malXY:cam*	[95]

### Antibiotics and fatty acids

All antibiotics were used at their standard concentrations (chloramphenicol 25;µg/ml, kanamycin 50 µg/ml, ampicillin 100 µg/ml) unless otherwise mentioned. c2-HDA was obtained from Cayman Chemicals (cat. 11133; used as the GC–MS standard and in all *in vitro hilA-lux* repression assays) and from Larodan (cat. 10-1609; used for growth-stimulation assays and selected *in vivo* experiments). Both sources were cross-validated against each other by GC–MS (matching retention time and fragment ions) and produced equivalent biological effects in pilot experiments. c11-Me-DDA was obtained from Sigma (cat. 42052).

### Construction of *Salmonella* mutant strains

Point mutations in *hilD* (*hilD* ^N44A^ and *hilD* ^Q290A^) were generated by allelic exchange as previously described^87^ using the suicide vector pCVD442^88^ (Addgene #11074), which carries the *sacB* gene conferring sucrose sensitivity in Gram-negative bacteria. Briefly, mutagenic primers (pB and pC) together with flanking primers (pA and pD) (listed in Table 2) were used to amplify two overlapping ~500 bp fragments of *hilD*. The overlapping PCR products were subsequently annealed and amplified using the flanking primers, generating a ~1 kb fragment containing the desired point mutation centrally located within the overlap region. The resulting fragment was cloned into pCVD442 using the restriction enzymes NdeI and AatII and transformed into a *pir* + *Escherichia coli* strain for plasmid maintenance and amplification. Transformants were selected on LB agar supplemented with ampicillin. The recombinant plasmid was then introduced into chloramphenicol-resistant *Salmonella* strain CA4434 (Table 1) by conjugation for 1 h at 37 °C, and transconjugants were selected on LB agar containing ampicillin and chloramphenicol. Integration of the plasmid at the *hilD* locus was confirmed by PCR. To select for plasmid excision, isolates were passaged on LB agar containing 5% sucrose. Candidate recombinants were screened by sequencing to identify clones that had lost the wild type *hilD* allele while retaining the desired point mutation.

**Table 2.** Primers used in this study.

Primer name	Sequence
invA-DW-FP	TGAAAAGCTGTCTTAATTTAATATTAACAGGATACCTATACCATGGTCCATATGAATATCCTCCTTAGTTC
invA-DW-RP	ATATCCAAATGTTGCATAGATCTTTTCCTTAATTAAGCCCGATTGTGTAGGCTGGAGCTGCTTCGAAGTTCCTAT
invA-chk-FP	TCACCAGCTCACCGTCTTTC
invA-chk-RP	GCTCCGCTGACCTACTGTTT
spiB-DW-FP	TAAAGCCTCAGTAGTAAATAATGGCATATCTCATGGTTAAGATTGTGTAGGCTGGAGCTGCTTCGAAGTTCCTAT
spiB-DW-RP	CAGCAAATCTTCTAACCGGGTCAAAGTTGTCATTTTCCACCCATGGTCCATATGAATATCCTCCTTAGTTC
spiB-chk-FP	TGGCTGCTATTGGGCGAA
spiB-chk-RP	GCCATTTTGAACCTGGGCAG
N44A-pA	CGACAGTAGTCATATGAATCGAACTATGTATGGCCCTGGG
N44A-pB	ACTGCTTACATAAAGAGCTTTGATGGTAACCTGC
N44A-pC	GCAGGTTACCATCAAAGCTCTTTATGTAAGCAGT
N44A-pD	GCACGTGCACGACGTCCGCGCTCCTTTAACGTTATCTGAGC
Q290A-pA	CGACAGTAGTCATATGTGGCGGTACCCACAGAGAAAGC
Q290A-pB	TTGAAACATGCAATGAAGTAGGACGTGC
Q290A-pC	GCACGTCCTACTTCATTGCATGTTTCAA
Q290A-pD	GCACGTGCACGACGTCGGCTGGCTCCAGGCATTATATCG
hilD-chk-FP	TAAGGAACATTAAAATAACATCAACAAAGGGATA
hilD-chk-RP	CATTGAATGAAGTAGGACGTGCTATC
gapA-RT-FP	GCGTCTAACAGGTCGTTGATTG
gapA-RT-RP	CTTCGTATTTGTCAAAGTTA
hilA-RT-FP	TCGTAGTGGTGTCTCCGCCAG
hilA-RT-RP	GGGGATTTTTGATAAACA
sopB-RT-FP	CCGATGCCTTAAGGCCAAA
sopB-RT-RP	AGAAGGCGTCTAACCACTCGCTGC
hilD-avrA-FP	GCTGGAAGGATTTCCTCTGGCAGGCAACCTTATAATTTCAATATACTGTTAGCGATGTCTGTCGTTC
hilD-avrA-RP	AACTAAGGAGGATATTCATATGGACCATGGTTACAGGGTGAAAAGTTATAATAACTAATACAAG
avrA-invH-hilD-chk-RP	ACCTAACCATGGAACATATCGTGC
sipD-pkd3-FP	GAGTCCTTACACTTGTAACCATTATTAATATCCTCTTCTGCATATGAATATCCTCCTTAG
sipD-pkd3-RP	GTGTGATTTAATCGCGCTCCTGATGGCGAACTGGGGATATGTGTAGGCTGGAGCTGCTTC
sipD-pkd3-chk-RP	CTGAAGGCGTCGATTCTCTG
rpfF FP	CAGCATAACCTTTTTCCGTCCTCATCGGGGGAATCTCCGT
rpfF RP	TTGGAGCTCCACCGCGGTGGGGCCGGGTCGCCAT
rpfF vector FP	GACGCAATGGCGACCCGGCCCCACCGCGGTGGAG
rpfF vector RP	ACGGAGATTCCCCCGATGAGGACGGAAAAAGGTTATGCTGCT
PrpfF-chk-FP	GCTGTTCGACCACCAGGG
PrpfF-chk-RP	GCCAGCTGGCGAAAGGGGGAT
Smalto-FOR	AAGGACAAGGCGATGACCATC
Smalto-REV	CCCCACCACGAYTTCATCA

*Salmonella* strains with deletions of *invA*, *spiB* and *sipD* were constructed using the *λ* Red recombination system^89^ as previously described.^17^ Using the primers (invA-DW-FP/RP, spiB-DW-FP/RP or sipD-pkd3-FP/RP listed in Table 2), these genes were replaced with chloramphenicol or kanamycin resistance genes using homologous recombination. Similarly, the ΔSPI1 *hilD* ^N44A^ strain was constructed by inserting a *hilD* ^N44A^-*cam* fragment into a ΔSPI1 mutant using *λ* Red recombination.^89^ The fragment was inserted between the *avr* and *invH* genes using primers containing homologous overhangs to those genes (Table 2). Loss of the genes and insertion of antibiotic cassettes were confirmed by PCR. Mutants were unmarked using the pCP20 recombinase method.^90^ P22 transduction was used to transfer genes among strains.^91^

### Construction of *S. maltophilia* Δ*rpfF* p*rpfF* complement strain

We constructed a plasmid containing the *rpfB* promoter and the *rpfF* ORF under *tetRA* promoter in a vector A086 (see Bacterial strains). We first constructed a plasmid by assembling *rpfFB* promoter into A086 vector and validated the construction by sequencing. We next assembled the *rpfF* ORF from *S. maltophilia* E77 genome after the *rpfB* promoter into the rpfB-A086 plasmid by Gibson assembly (NEB). The assembly was verified by check PCR using the primers prpfF-chk-FP/RP (Table 2). Confirmed plasmid was electroporated into *S. maltophilia* Δ*rpfF.*

### Murine *Salmonella* infections

The experiments in Figure 1 were performed as described here, using the 129X1/SvJ mouse model. This is a well-established model that is susceptible to *Salmonella* colonization without the use of antibiotics and has been used for long-term infections.^46^ Female 6–8 week old mice were procured from Jackson Laboratories. *Salmonella* strains were competed by administering equal numbers of each strain (wild type and mutant) by oral gavage. A total of 1 × 10^8^ CFU for both strains in a competition assay were administered in 100 µL PBS. Fecal pellets were collected for plating to assess colonization. Inflammation was tested by fecal lipocalin assay (R&D systems). Competitive indices were calculated by correcting the ratio of fecal CFU counts with that of the inoculum.

### *S. maltophilia* mouse model

Female 6–8 weeks old 129X1/SvJ mice were procured from Jackson Laboratories. On day 0, fecal samples were collected, and mice were orally gavaged with 0.1 ml streptomycin solution (200 mg/ml) for partial gut clearance. On day 1, food was withheld from all groups for one hour followed by oral gavage of 0.1 ml 1% w/v sodium bicarbonate solution. This was immediately followed by oral gavage of 0.1 ml solution of either sterile PBS or 1 × 10^6^ CFU *S. maltophilia* wild type or the Δ*rpfF* mutant strain. This procedure was repeated on day 2. Mice were observed on day 3–5 and fecal pellets were collected. *S. maltophilia* colonization was monitored by plating the fecal samples on VIA agar plates. Colonic inflammation was measured by fecal lipocalin ELISA (R&D systems) of fecal samples.

Fecal pellets collected throughout the experiment were used for DNA extraction using the Qiagen PowerFood DNA Isolation Kit according to the manufacturer's instructions. Extracted DNA was quantified and normalized to 75 ng per reaction for use as template for qPCR. Quantitative PCR was performed using the Bio-Rad iTaq Universal One-Step RT-qPCR Kit, omitting the reverse transcription step. Amplification was carried out using Smalto-FOR/REV primers (Table 2), which target a conserved region of the *fdnG* gene encoding formate dehydrogenase, as previously described.^92^ Genomic DNA standards were used to determine the limit of detection (100 fg) and assess false positive rates. For each time point, three biological replicates and three technical replicates were included, for a total of nine replicates per condition.

### *Salmonella* infection in the *S. maltophilia* mouse model

Female 129X1/SvJ mice (6–8 weeks old) were orally gavaged with streptomycin followed by *S. maltophilia* as described above. On day 3, all mice were infected with 1 × 10^5^ CFU *Salmonella* (wild type or *hilD* ^N44A^) in 0.1 ml PBS, each marked with distinct antibiotic resistance cassettes. On day 5 all mice were euthanized, and ileum, cecum and colon sections were collected for histopathology. Colon contents were collected and plated on LB agar with the appropriate antibiotic to quantify *Salmonella*. Throughout the experiment, fecal pellets were collected and spread on VIA agar plates to quantify *S. maltophilia*.

### Histology of murine intestine and scoring

On day 5, after euthanasia, distal ileum, cecum and proximal colon of mice were collected and fixed in 10% neutral-buffered formalin, embedded in paraffin, sectioned, and stained with hematoxylin and eosin (H&E). Histopathologic changes of the cecum were evaluated blindly by a board-certified pathologist (RCC) and scored with a semiquantitative scale modified from Barthel M. et al.^51^ Evaluated criteria include the degree of submucosal edema, severity of neutrophilic infiltration, number of goblet cells, and epithelial integrity. The details of the scoring process are described in the Supplementary methods.

### Fecal lipocalin ELISA

These experiments were performed using the mouse lipocalin ELISA kit from R&D systems, following their protocol. Briefly, fecal samples were weighed and homogenized in one ml PBS by vortexing rigorously. ELISA plates were coated with capture antibody and incubated overnight. The next day, the capture antibody was washed, and the plate was blocked with assay diluent. After incubation, standard lipocalin solutions or 0.1 ml of fecal samples were added and incubated at room temperature for two hours. After washing the plate, detection antibody was added, and the plate was incubated at room temperature for one hour. After washing, substrate was added and incubated until the development of color. The reaction was stopped by adding stop solution and the color was read at 450 nm in a spectrophotometer. A standard curve was computed based on the different concentrations of the standard lipocalin solutions. The concentrations of lipocalin in the fecal samples were calculated based on the standard curve.

### Fatty acid extraction

To extract fatty acids from the culture supernatants of *S. maltophilia* and murine colon contents, the Folch method was followed.^93^ Cultures were grown with various treatments in MGAM media for 12 h at 30 °C. Cultures were centrifuged and the supernatant was collected. The Folch reagent (2:1 chloroform:methanol) in 20-fold excess was added according to the culture volume. After brief incubation, 4-fold excess culture volume of water was added and mixed until the phases separated. The bottom phase was collected and evaporated under air flow. Extracts were resuspended in 1 ml ethanol. For mouse experiments, the colon contents were resuspended into 1 ml of sterile PBS and vortexed rigorously to homogenize. These were then passed through a 0.45 micron filter and the final filtrate volume was measured. The Folch reagent was added in 20-fold excess volume of the filtrate and stirred with a glass rod rigorously. After brief incubation, water was added in 4-fold excess volume of the filtrate and mixed until the phases separated. The bottom phase was collected and evaporated under air flow. Extracts were resuspended in 1 ml ethanol for bacterial cultures and 0.3 ml for mouse gut contents, and analyzed by GC–MS.

### Gene expression/luciferase assays

*Salmonella* strains carrying the *hilA-luxCDABE* reporter plasmid were grown overnight with 1 mM nonanoic acid (to repress background SPI-1 expression) and chloramphenicol (25 µg/ml). Final cultures were washed with PBS and resuspended in LB buffered with 100 mM MOPS (3-morpholinopropane-1-sulfonic acid, pH 6.7), with 25 µg/ml chloramphenicol. Bacteria were inoculated at a starting OD600 of 0.02 into 150 µl of the same media with 10 µl solvent control (ethanol) or fatty acid extracts. c2-HDA at 2 µM concentration was used as control. Luminescence and absorbance were read every half hour for 24 h in Biotek Synergy H1 microplate reader. Data were analyzed in Microsoft Excel and Graphpad Prism. Area Under Curves (AUC) for each condition were generated. AUC of solvent control (ethanol) was normalized to 100% and others were calculated accordingly.

### RNA isolation, cDNA synthesis, and RT-qPCR

The expression of *hilA* and *sopB* in *Salmonella* wild type and *hilD* ^N44A^ mutants recovered from the colon contents was analyzed by RT-qPCR. Colon contents were diluted in 10 mg/ml PBS and passed through 0.45 micron filter to remove debris. Bacteria were collected by centrifugation at 10,000 x *g* for 10 min. Supernatant was discarded and the pellet was resuspended in 1 ml TRIzol (Invitrogen) and incubated for five minutes on ice. Total RNA was extracted by following the TRIzol Reagent user guide. iTaq Universal SYBR Green One-Step Kit (Bio-Rad) was used to transcribe the RNA into a complementary DNA template and perform quantitative PCR in a single reaction mix using primers *gapA*-RT-FP/RP, *hilA*-RT-FP/RP, and *sopB*-RT-FP/RP. Relative expression of *hilA* and *sopB* were calculated by using the threshold cycle (∆∆Ct) method^94^ relative to the housekeeping gene *gapA*.

### Gas chromatography mass spectrometry (GC–MS)

To identify the specific fatty acid species in the *S. maltophilia* culture supernatants and murine colon contents, fatty acid extracts were subjected to GC–MS analysis. A volume of 0.05 ml extract in ethanol was esterified overnight in 0.2 ml of a 1:1 mixture of hexane and 3-(Trifluoromethyl) phenyltrimethylammonium hydroxide (TCI chemicals). The samples were analyzed in the Thermo Scientific Exactive GC-Orbitrap GC–MS/MS on an Agilent Ultra 2 column (19091B-102). Fatty acid methyl esters were injected in split mode with a split ratio of 50:1. The injector temperature was set to 250 °C. The oven temperature program was as follows: initial temperature of 100 °C was held for 2 min, increased at 15 °C per min to 150 °C, held for 4 min, increased at 10 °C per min to 280 °C, where it was held for the remainder of the 30 min run. The mass spectrometer was operated in electron ionization (EI) mode at 70 eV. The ion source temperature was set to 280 °C, and the MS transfer line temperature was maintained at 250 °C. Data were acquired in full scan mode (Full MS) in positive ion polarity over an *m/z* range of 35–550, with a resolving power of 60,000 (at *m/z* 200). Characteristic retention times and fragmentation patterns were compared against the c2-HDA standard for confirmation. Spectra were analyzed using FreeStyle 1.8 SP.

### Stimulation with different NO donors

To distinguish whether nitric oxide (NO) or peroxynitrite (ONOO⁻) is the primary nitrogen-derived reactive species that induces c2-HDA production by *S. maltophilia*, cultures were treated separately with the selective NO donor DN (Cayman Chemicals, 82120) and with the peroxynitrite donor SIN (Cayman Chemicals, 82220). Stock solutions of DN (100 mM) were prepared fresh in 10 mM NaOH to maintain alkaline pH and prevent premature decomposition, aliquoted, and used immediately. SIN stock solutions (100 mM) were prepared fresh in ice-cold deoxygenated 1 × PBS (pH 7.4) immediately before each experiment to limit decomposition and were kept on ice and protected from light until added to cultures. *S. maltophilia* wild type, Δ*rpfF* or Δ*rpfF* p*rpfF* strains were grown overnight in MGAM medium at 30 °C, diluted to a starting OD600 of 0.05 in fresh MGAM, and treated with DN or SIN1 at final concentrations of 100, 250, and 500 µM. Parallel cultures received an equivalent volume of vehicle (10 mM NaOH for DN; 1 × PBS for SIN), and decomposed-donor controls (DN or SIN pre-incubated in MGAM at 37 °C for 24 h before addition to the culture, to allow exhaustive NO release) were included to confirm that the observed effects are dependent on active donor decomposition rather than on residual donor chemistry. Cultures were incubated at 30 °C with aeration for 12 h, after which cells were pelleted by centrifugation and the supernatants were collected for fatty acid extraction using the Folch method, as described above.

To verify the chemical species generated under each treatment, a parallel set of cell-free MGAM samples containing each donor was assayed in 96-well plates. NO production was quantified by the Greiss reagent assay (Thermo Fisher) as described above. Peroxynitrite formation was assessed by the oxidation of dihydrorhodamine 123 (DHR123; Invitrogen, D632) to the fluorescent product rhodamine 123. Briefly, 0.05  ml of donor-containing or vehicle-containing MGAM was mixed with 0.05 ml of 50 µM DHR123 in 1 × PBS, incubated at 37 °C for 30 min in the dark, and fluorescence was measured at excitation 485 nm and emission 528 nm using a Biotek Synergy H1 microplate reader. Authentic peroxynitrite (Cayman Chemicals, 81565) at 1–25 µM was used as a standard. As a complementary control, parallel cultures were co-treated with the selective peroxynitrite scavenger FeTPPS (5,10,15,20-tetrakis (4-sulfonatophenyl) porphyrinato iron(III); Cayman Chemicals, 17187) at 50 µM, which is expected to abolish the SIN-driven response without affecting the DN-driven response. All experiments were performed at least twice independently with five biological replicates per condition.

### Macrophage stimulation and co-culture assays

THP-1 monocytes (ATCC: TIB202) were maintained in RPMI-1640 supplemented with 10% FBS in T75 (NUNC) flasks in 5% CO_2_ incubator at 37 °C. Prior to experiments, cells were counted and seeded at ~10^5^/ml media in 24-well tissue culture plates (NUNC) and differentiated with 1 µM PMA for 16 h. Afterwards, media was replaced with fresh RPMI containing 10% FBS and incubated for 16 h. To generate inflammation, these THP-1 macrophages were either stimulated with 100 ng/mL LPS for 12 h or infected with *Salmonella* (multiplicity of infection, MOI of 10:1) wild type or mutant strains for 4 h. After incubation, the cell culture supernatants were collected and filtered (0.22 µm). Aliquots were separated for Greiss and Amplex Red assay to measure RNS amounts. The remaining volume was utilized for growth of *S. maltophilia*. For antioxidant experiments, Trolox (100 µM) was added to the THP-1 macrophage supernatants and incubated for two hours. These were then used to grow *S. maltophilia* to analyze the effect on the expression of fatty acids.

### Assessment of RNS production by Greiss reagent

Cell culture supernatants were collected, and RNS levels were determined using the Greiss reagent (Thermo Fisher) as per manufacturer's protocol. Briefly, 0.13 ml culture supernatant was mixed 0.02 ml of Greiss reagent and 0.15 ml deionized water and incubated at room temperature for 30 min. The absorbance of the mixture was measured at 548 nm using Biotek Synergy H1 Microplate Reader. Standards of NO solutions were used to construct a standard curve and were used to calculate the amounts of RNS in the culture supernatants.

### Assessment of ROS production by Amplex red assay

ROS production was quantified in macrophage culture supernatants using the Amplex Red Hydrogen Peroxide/Peroxidase Assay Kit (Invitrogen/Thermo Fisher, A22188) following the manufacturer's protocol. Briefly, THP-1 macrophage culture supernatants were collected immediately following stimulation (12 h with 100 ng/mL LPS, or 4 h with *Salmonella* wild type or Δ*invA* Δ*spiB* mutant strains at MOI 10:1) and filtered through a 0.22 µm membrane to remove cellular debris and bacteria. A working solution containing 100 µM Amplex Red reagent and 0.2 U/mL horseradish peroxidase (HRP) were freshly prepared in 1 × reaction buffer (50 mM sodium phosphate, pH 7.4). In a black-walled, clear-bottom 96-well plate (NUNC), 0.05 ml of culture supernatant was mixed with 0.05 ml of the Amplex Red/HRP working solution. Wells receiving 1 × reaction buffer in place of supernatant served as background controls. The plate was incubated at room temperature for 30 min in the dark. Fluorescence was then read in a Biotek Synergy H1 microplate reader at excitation 540 nm and emission 590 nm. A standard curve was generated in parallel using serial dilutions of HP prepared fresh in 1 × reaction buffer from a 30% HP stock (Sigma; 1009). Background fluorescence from the reagent-only control was subtracted from all sample and standard readings. HP concentrations in the macrophage supernatants were interpolated from the linear range of the standard curve. For experiments assessing antioxidant quenching, Trolox (100 µM) was added to macrophage supernatants and incubated for 2 h at 37 °C prior to Amplex Red measurement. All samples were assayed in technical triplicate, and the experiment was performed independently at least twice with five biological replicates per condition.

### Bacterial growth assays

To investigate whether c2-HDA influences *Salmonella* proliferation, overnight cultures of wild type or mutant *Salmonella* were diluted to OD600 = 0.05 in fresh LB medium with or without fatty acid extracts. OD600 readings were recorded every 30 min for 24 h in Biotek Synergy H1 microplate reader.

### Statistical analysis

All *in vitro* experiments were carried out at least twice with five technical replicates; each mouse infection study was performed once, with 5 mice/group. Data are presented as means ± SD. Statistical comparisons were conducted in GraphPad Prism 10 using nonparametric, unpaired *t*-tests. Differences were considered significant when *p* < 0.05 (indicated by *), *p* < 0.01 (indicated by **), and *p* < 0.001 (indicated by ***). ns is not significant.

## Supplementary Material

Supplementary Material Upload Final Supplementary Figures 06112026.docx

Supplementary Material Supplementary Methods Mar 1.docx

## Data Availability

All raw data can be found at “Chowdhury, Rimi (2026), *Salmonella* exploits a quorum-sensing family signal of the gut commensal *S. maltophilia* to facilitate its colonization, Mendeley Data, V1, doi: 10.17632/y33bgc6cv4.1”.

## Resource availability

Resources and further information can be requested to the lead contacts Dr. Rimi Chowdhury (rchowdhury@miamioh.edu) and Dr. Craig Altier (altier@cornell.edu).

## References

[1] Yang J , Tauschek M , Hart E , Hartland EL , Robins-Browne RM . Virulence regulation in citrobacter rodentium: the art of timing. Microb Biotechnol. 2010;3:259–268. doi: 10.1111/j.1751-7915.2009.00114.x. 21255326 PMC3815369

[2] Liu Y , Han R , Wang J , Yang P , Wang F , Yang B . Magnesium sensing regulates intestinal colonization of enterohemorrhagic escherichia coli O157:H7. mBio. 2020;11:e02470-20. doi: 10.1128/mBio.02470-20. 33173003 PMC7667037

[3] Yang B , Feng L , Wang F , Wang L . Enterohemorrhagic escherichia coli senses low biotin status in the large intestine for colonization and infection. Nat Commun. 2015;6:6592. doi: 10.1038/ncomms7592. 25791315 PMC4382993

[4] Rothenbacher FP , Zhu J . Efficient responses to host and bacterial signals during vibrio cholerae colonization. Gut Microbes. 2014;5:120–128. doi: 10.4161/gmic.26944. 24256715 PMC4049929

[5] De Nisco NJ , Rivera-Cancel G , Orth K . The biochemistry of sensing: enteric pathogens regulate type III secretion in response to environmental and host cues. mBio. 2018;9:e02122-17. doi: 10.1128/mBio.02122-17. 29339429 PMC5770552

[6] Sturm A , Heinemann M , Arnoldini M , Benecke A , Ackermann M , Benz M , Dormann J , Hardt W-D . The cost of virulence: retarded growth of salmonella typhimurium cells expressing type III secretion system 1. PLoS Pathog. 2011;7:e1002143. doi: 10.1371/journal.ppat.1002143. 21829349 PMC3145796

[7] Sassone-Corsi M , Raffatellu M . No vacancy: how beneficial microbes cooperate with immunity to provide colonization resistance to pathogens. J Immunol. 2015;194:4081–4087. doi: 10.4049/jimmunol.1403169. 25888704 PMC4402713

[8] Bäumler AJ , Sperandio V . Interactions between the microbiota and pathogenic bacteria in the gut. Nature. 2016;535:85–93. doi: 10.1038/nature18849. 27383983 PMC5114849

[9] Altier C . Starving out the enemy. Cell Host Microbe. 2020;28:501–502. doi: 10.1016/j.chom.2020.09.003. 33031733

[10] Jones BD . Salmonella invasion gene expression: a story of environmental awareness. J Microbiol. 2005:110–117. 15765064

[11] Stecher B , Robbiani R , Walker AW , Westendorf AM , Barthel M , Kremer M , Chaffron S , Macpherson AJ , Buer J , Parkhill J , et al. Salmonella enterica serovar typhimurium exploits inflammation to compete with the intestinal microbiota. PLoS Biol. 2007;5:2177–2189. doi: 10.1371/journal.pbio.0050244. 17760501 PMC1951780

[12] Huang Y , Suyemoto M , Garner CD , Cicconi KM , Altier C . Formate acts as a diffusible signal to induce salmonella invasion. J Bacteriol. 2008;190:4233–4241. doi: 10.1128/JB.00205-08. 18424519 PMC2446767

[13] Rivera-Chávez F , Winter SE , Lopez CA , Xavier MN , Winter MG , Nuccio S-P , Russell JM , Laughlin RC , Lawhon SD , Sterzenbach T , et al. Salmonella uses energy taxis to benefit from intestinal inflammation. PLoS Pathog. 2013;9:e1003267. doi: 10.1371/journal.ppat.1003267. 23637594 PMC3630101

[14] Galán JE . Salmonella typhimurium and inflammation: a pathogen-centric affair. Nat Rev Microbiol. 2021;19:716–725. doi: 10.1038/s41579-021-00561-4. 34012042 PMC9350856

[15] Hung C-C , Garner CD , Slauch JM , Dwyer ZW , Lawhon SD , Frye JG , McClelland M , Ahmer BMM , Altier C . The intestinal fatty acid propionate inhibits salmonella invasion through the post-translational control of HilD. Mol Microbiol. 2013;87:1045–1060. doi: 10.1111/mmi.12149. 23289537 PMC3581741

[16] Golubeva YA , Ellermeier JR , Cott Chubiz JE , Slauch JM . Intestinal long-chain fatty acids act as a direct signal to modulate expression of the salmonella pathogenicity island 1 type III secretion system. mBio. 2016;7:e02170–02115. doi: 10.1128/mBio.02170-15. 26884427 PMC4752608

[17] Bosire EM , Eade CR , Schiltz CJ , Reid AJ , Troutman J , Chappie JS , Altier C . Diffusible signal factors act through AraC-Type transcriptional regulators as chemical cues to repress virulence of enteric pathogens. Infect Immun. 2020;88:e00226-20. doi: 10.1128/IAI.00226-20. 32690633 PMC7504960

[18] Chowdhury R , Pavinski Bitar PD , Adams MC , Chappie JS , Altier C . AraC-type regulators HilC and RtsA are directly controlled by an intestinal fatty acid to regulate salmonella invasion. Mol Microbiol. 2021;116:1464–1475. doi: 10.1111/mmi.14835. 34687258 PMC8688230

[19] Chowdhury R , Pavinski Bitar PD , Keresztes I , Condo AM , Altier C . A diffusible signal factor of the intestine dictates salmonella invasion through its direct control of the virulence activator HilD. PLoS Pathog. 2021;17:e1009357. doi: 10.1371/journal.ppat.1009357. 33617591 PMC7932555

[20] Chowdhury R , Pavinski Bitar PD , Bell KE , Altier C . Shigella flexneri utilizes intestinal signals to control its virulence. Gut Microbes. 2023;15. doi: 10.1080/19490976.2023.2256767. PMC10519361 37741806

[21] Prouty AM , Gunn JS . Salmonella enterica serovar typhimurium invasion is repressed in the presence of bile. Infect Immun. 2000;68:6763–6769. doi: 10.1128/IAI.68.12.6763-6769.2000. 11083793 PMC97778

[22] Eade CR , Hung C-C , Bullard B , Gonzalez-Escobedo G , Gunn JS , Altier C . Bile acids function synergistically to repress invasion gene expression in salmonella by destabilizing the invasion regulator HilD. Infect Immun. 2016;84:2198–2208. doi: 10.1128/IAI.00177-16. 27185788 PMC4962646

[23] Chowdhury R , Pavinski Bitar PD , Chapman HM , Altier C . Salmonella invasion is controlled by competition among intestinal chemical signals. mBio. 2023;14:e00012-23. doi: 10.1128/mbio.00012-23. 37017539 PMC10127606

[24] Lucas RL , Lee CA . Roles of hilC and hilD in regulation of hilA expression in salmonella enterica serovar typhimurium. J Bacteriol. 2001;183:2733–2745. doi: 10.1128/JB.183.9.2733-2745.2001. 11292791 PMC99488

[25] Ellermeier CD , Ellermeier JR , Slauch JM . HilD, HilC and RtsA constitute a feed forward loop that controls expression of the SPI1 type three secretion system regulator hilA in salmonella enterica serovar typhimurium. Mol Microbiol. 2005;57:691–705. doi: 10.1111/j.1365-2958.2005.04737.x. 16045614

[26] Ellermeier JR , Slauch JM . Adaptation to the host environment: regulation of the SPI1 type III secretion system in salmonella enterica serovar typhimurium. Curr Opin Microbiol. 2007;10:24–29. doi: 10.1016/j.mib.2006.12.002. 17208038

[27] Golubeva YA , Sadik AY , Ellermeier JR , Slauch JM . Integrating global regulatory input into the salmonella pathogenicity island 1 type III secretion system. Genetics. 2012;190:79–90. doi: 10.1534/genetics.111.132779. 22021388 PMC3249375

[28] Chowdhury R , Bitar PDP , Bell KE , Altier C . Shigella flexneri utilizes intestinal signals to control its virulence. Gut Microbes. 2023;15:2256767. doi: 10.1080/19490976.2023.2256767. 37741806 PMC10519361

[29] Mitchell MK , Ellermann M . Long chain fatty acids and virulence repression in intestinal bacterial pathogens. Front Cell Infect Microbiol. 2022;12:928503. doi: 10.3389/fcimb.2022.928503. 35782143 PMC9247172

[30] Borreby C , Lillebæk EMS , Kallipolitis BH . Anti-infective activities of long-chain fatty acids against foodborne pathogens. FEMS Microbiol Rev. 2023;47:fuad037. doi: 10.1093/femsre/fuad037. 37437907 PMC10368373

[31] Trirocco R , Pasqua M , Tramonti A , Grossi M , Colonna B , Paiardini A , Prosseda G , et al. Fatty acids abolish shigella virulence by inhibiting its master regulator, VirF. Microbiology Spectrum. 2023;11:e00778–23. 37140433 10.1128/spectrum.00778-23 PMC10269687

[32] Bosire EM , Adams MC , Pavinski Bitar PD , Murphy SG , Shin J-H , Chappie JS , Dörr T , Altier C . Vibrio cholerae integrates interspecies quorum-sensing signals to regulate virulence. mBio. 2025;0:e01537-25 10.1128/mbio.01537-25 PMC12421807 40742133

[33] Beaulieu ED , Ionescu M , Chatterjee S , Yokota K , Trauner D , Lindow S . Characterization of a diffusible signaling factor from *Xylella fastidiosa* . mBio. 2013;4:e00539–00512. doi: 10.1128/mBio.00539-12. 23300249 PMC3546559

[34] Ryan RP , An S , Allan JH , McCarthy Y , Dow JM . The DSF family of cell-cell signals: an expanding class of bacterial virulence regulators. PLoS Pathog. 2015;11:e1004986. doi: 10.1371/journal.ppat.1004986. 26181439 PMC4504480

[35] Ionescu M , Yokota K , Antonova E , Garcia A , Beaulieu E , Hayes T , Iavarone AT , Lindow SE . Promiscuous diffusible signal factor production and responsiveness of the *Xylella fastidiosa* rpf system. mBio. 2016;7:e01054-16. doi: 10.1128/mBio.01054-16. 27435463 PMC4958263

[36] He Y-W , Deng Y , Miao Y , Chatterjee S , Tran TM , Tian J , Lindow S . DSF-family quorum sensing signal-mediated intraspecies, interspecies, and inter-kingdom communication. TIM. 2023;31:36–50. doi: 10.1016/j.tim.2022.07.006. 35941062

[37] Rather MA , Chowdhury R , Pavinski Bitar PD , Altier C . Recombinant production of a diffusible signal factor inhibits salmonella invasion and animal carriage. Gut Microbes. 2023;15:2208498. doi: 10.1080/19490976.2023.2208498. 37158497 PMC10171134

[38] Yang J , Pu J , Lu S , Bai X , Wu Y , Jin D , Cheng Y , Zhang G , Zhu W , Luo X , et al. Species-level analysis of human gut microbiota with metataxonomics. Front Microbiol. 2020;11. [cited 2025 May 30]. Available from: https://www.frontiersin.org/journals/microbiology/articles/10.3389/fmicb.2020.02029/full. 10.3389/fmicb.2020.02029 PMC7479098 32983030

[39] Leviatan S , Shoer S , Rothschild D , Gorodetski M , Segal E . An expanded reference map of the human gut microbiome reveals hundreds of previously unknown species. Nat Commun. 2022;13:3863. doi: 10.1038/s41467-022-31502-1. 35790781 PMC9256738

[40] Pédron T , Mulet C , Dauga C , Frangeul L , Chervaux C , Grompone G , Sansonetti PJ . A crypt-specific core microbiota resides in the mouse colon. mBio. 2012;3. doi:10.1128/mbio.00116-12. PMC3372965 22617141

[41] Saffarian A , Mulet C , Regnault B , Amiot A , Tran-Van-Nhieu J , Ravel J , Sobhani I , Sansonetti PJ , Pédron T . Crypt- and mucosa-associated core microbiotas in humans and their alteration in colon cancer patients. mBio. 2019;10. doi: 10.1128/mBio.01315-19. PMC6635529 31311881

[42] Wang M , Qazi IH , Wang L , Zhou G , Han H . Salmonella virulence and immune escape. Microorganisms. 2020;8:407. doi: 10.3390/microorganisms8030407. 32183199 PMC7143636

[43] Huedo P , Yero D , Martínez-Servat S , Ruyra À , Roher N , Daura X , Gibert I . Decoding the genetic and functional diversity of the DSF quorum-sensing system in *Stenotrophomonas maltophilia* . Front Microbiol [Internet]. 2015;6. doi: 10.3389/fmicb.2015.00761. cited 2025 May 24]. Available from: https://www.frontiersin.org/journals/microbiology/articles/10.3389/fmicb.2015.00761/full. PMC4517397 26284046

[44] Dow JM . Diffusible signal factor-dependent quorum sensing in pathogenic bacteria and its exploitation for disease control. J Appl Microbiol. 2017;122:2–11. doi: 10.1111/jam.13307. 27684652

[45] Zhou L , Zhang L-H , Cámara M , He Y-W . The DSF family of quorum sensing signals: diversity, biosynthesis, and turnover. Trends Microbiol. 2017;25:293–303. doi: 10.1016/j.tim.2016.11.013. 27979499

[46] Lawley TD , Bouley DM , Hoy YE , Gerke C , Relman DA , Monack DM . Host transmission of salmonella enterica serovar typhimurium is controlled by virulence factors and indigenous intestinal microbiota. Infect Immun. 2007;76:403. 17967858 10.1128/IAI.01189-07 PMC2223630

[47] Gül E , Bakkeren E , Salazar G , Steiger Y , Younes AA , Clerc M , Christen P , Fattinger SA , Nguyen BD , Kiefer P , et al. The microbiota conditions a gut milieu that selects for wild-type salmonella typhimurium virulence. PLoS Biol. 2023;21:e3002253. doi: 10.1371/journal.pbio.3002253. 37651408 PMC10499267

[48] An S , Tang J . Diffusible signal factor signaling regulates multiple functions in the opportunistic pathogen *Stenotrophomonas maltophilia* . BMC Res Notes. 2018;11:569. doi: 10.1186/s13104-018-3690-1. 30097057 PMC6086056

[49] Huang TP . Biofilm formation and cell-cell signaling in *Stenotrophomonas maltophilia* . 2007. Madison: University of Wisconsin-Madison Ph.D. thesis.

[50] Takahashi I , Hosomi K , Nagatake T , Toubou H , Yamamoto D , Hayashi I , Kurashima Y , Sato S , Shibata N , Goto Y , et al. Persistent colonization of non-lymphoid tissue-resident macrophages by *Stenotrophomonas maltophilia* . Int Immunol. 2020;32:133–141. doi: 10.1093/intimm/dxz071. 31630178 PMC10689348

[51] Barthel M , Hapfelmeier . Pretreatment of mice with streptomycin provides a salmonella enterica serovar typhimurium colitis model that allows analysis of both pathogen and host. Infect Immun. 2003;71:2839–2858. doi: 10.1128/IAI.71.5.2839-2858.2003. 12704158 PMC153285

[52] Whitaker WB , Parent MA , Boyd A , Richards GP , Boyd EF . The vibrio parahaemolyticus ToxRS regulator is required for stress tolerance and colonization in a novel orogastric streptomycin-induced adult murine model. Infect Immun. 2012;80:1834–1845. doi: 10.1128/IAI.06284-11. [cited 2025 Dec 21]. Available from: https://journals.asm.org/doi/10.1128/iai.06284-11. 22392925 PMC3347455

[53] Alphonse N , Odendall C . Animal models of shigellosis: a historical overview. Curr Opin Immunol. 2023;85:102399. doi: 10.1016/j.coi.2023.102399. 37952487

[54] Miller CP , Bohnhoff M . Changes in the mouse’s enteric microflora associated with enhanced susceptibility to salmonella infection following streptomycin treatment*. J Infect Dis. 1963;113:59–66. doi: 10.1093/infdis/113.1.59. 14044094

[55] Keefer LK , Nims RW , Davies KM , Wink DA . NONOates” (1-substituted diazen-1-ium-1,2-diolates) as nitric oxide donors: convenient nitric oxide dosage forms. Methods Enzymol. 1996;268:281–293. doi: 10.1016/S0076-6879(96)68030-6. 8782594

[56] Achary AS , Mahapatra C . Reactive nitrogen species-mediated cell proliferation during tail regeneration and retinoic acid as a putative modulator of tissue regeneration in the geckos. Cells Dev. 2024;177:203901. doi: 10.1016/j.cdev.2024.203901. 38278363

[57] Aviello G , Knaus U . ROS in gastrointestinal inflammation: rescue or sabotage? Br J Pharmacol. 2017;174:1704–1718. doi: 10.1111/bph.13428. 26758851 PMC5446568

[58] Ryan KA , Smith MF , Sanders MK , Ernst PB . Reactive oxygen and nitrogen species differentially regulate toll-like receptor 4-Mediated activation of NF-κB and Interleukin-8 expression. Infect Immun. 2004;72:2123–2130. doi: 10.1128/IAI.72.4.2123-2130.2004. 15039334 PMC375203

[59] Huang D , Ou B , Prior RL . The chemistry behind antioxidant capacity assays. J Agric Food Chem. 2005;53:1841–1856. doi: 10.1021/jf030723c. 15769103

[60] Salgo MG , Pryor WA . Trolox inhibits peroxynitrite-mediated oxidative stress and apoptosis in rat thymocytes. Arch Biochem Biophys. 1996;333:482–488. doi: 10.1006/abbi.1996.0418. 8809090

[61] Bisby RH , Parker AW . Reaction of ascorbate with the alpha-tocopheroxyl radical in micellar and bilayer membrane systems. Arch Biochem Biophys. 1995;317:170–178. doi: 10.1006/abbi.1995.1150. 7872780

[62] Vaya J , Aviram M . Nutritional antioxidants mechanisms of action, analyses of activities and medical applications. Curr Med Chem - Immunol, Endocr Metab Agents. 2001;1:99–117. doi: 10.2174/1568013013359168.

[63] Bao H , Wang S , Zhao J-H , Liu S-L . *Salmonella* secretion systems: differential roles in pathogen-host interactions. Microbiol Res. 2020;241:126591. doi: 10.1016/j.micres.2020.126591. 32932132

[64] Liou MJ , Miller BM , Litvak Y , Nguyen H , Natwick DE , Savage HP , Rixon JA , Mahan SP , Hiyoshi H , Rogers AWL , et al. Host cells subdivide nutrient-niches into discrete biogeographical microhabitats for gut microbes. Cell Host Microbe. 2022;30:836–847.e6. doi: 10.1016/j.chom.2022.04.012. 35568027 PMC9187619

[65] Proctor LM , Creasy HH , Fettweis JM , Lloyd-Price J , Mahurkar A , Zhou W , Buck GA , Snyder MP , Strauss JF , Weinstock GM , et al. The integrative human microbiome project. Nature. 2019;569:641–648. doi: 10.1038/s41586-019-1238-8. 31142853 PMC6784865

[66] Zheng D , Liwinski T , Elinav E . Interaction between microbiota and immunity in health and disease. Cell Res. 2020;30:492–506. doi: 10.1038/s41422-020-0332-7. 32433595 PMC7264227

[67] Ferreyra JA , Wu KJ , Hryckowian AJ , Bouley DM , Weimer BC , Sonnenburg JL . Gut microbiota-produced succinate promotes C. Difficile infection after antibiotic treatment or motility disturbance. Cell Host Microbe. 2014;16:770–777. 25498344 10.1016/j.chom.2014.11.003 PMC4859344

[68] Ng KM , Ferreyra JA , Higginbottom SK , Lynch JB , Kashyap PC , Gopinath S , Naidu N , Choudhury B , Weimer BC , Monack DM , et al. Microbiota-liberated host sugars facilitate post-antibiotic expansion of enteric pathogens. Nature. 2013;502:96–99. doi: 10.1038/nature12503. 23995682 PMC3825626

[69] Pickard JM , Zeng MY , Caruso R , Núñez G . Gut microbiota: role in pathogen colonization, immune responses and inflammatory disease. Immunol Rev. 2017;279:70–89. doi: 10.1111/imr.12567. 28856738 PMC5657496

[70] Ashida H , Ogawa M , Mimuro H , Kobayashi T , Sanada T , Sasakawa C . *Shigella* are versatile mucosal pathogens that circumvent the host innate immune system. Curr Opin Immunol. 2011;23:448–455. doi: 10.1016/j.coi.2011.06.001. 21763117

[71] Ashida H , Mimuro H , Sasakawa C . Shigella manipulates host immune responses by delivering effector proteins with specific roles. Front Immunol. 2015;6. doi:10.3389/fimmu.2015.00219. [cited 2025 May 23]. Available from: https://www.frontiersin.org/journals/immunology/articles/10.3389/fimmu.2015.00219/full. PMC4423471 25999954

[72] Lopez CA , Miller BM , Rivera-Chávez F , Velazquez EM , Byndloss MX , Chávez-Arroyo A , Lokken KL , Tsolis RM , Winter SE , Bäumler AJ . Virulence factors enhance *citrobacter rodentium* expansion through aerobic respiration. Science. 2016;353:1249–1253. doi: 10.1126/science.aag3042. 27634526 PMC5127919

[73] Spees AM , Wangdi T , Lopez CA , Kingsbury DD , Xavier MN , Winter SE , Tsolis RM , Bäumler AJ . Streptomycin-induced inflammation enhances escherichia coli gut colonization through nitrate respiration. mBio. 2013;4, 10.1128/mbio.00430-13. PMC3705454 23820397

[74] Winter SE , Winter MG , Xavier MN , Thiennimitr P , Poon V , Keestra AM , Laughlin RC , Gomez G , Wu J , Lawhon SD , et al. Host-derived nitrate boosts growth of *e. coli* in the inflamed gut. Science. 2013;339:708–711. doi: 10.1126/science.1232467. 23393266 PMC4004111

[75] Winter SE , Thiennimitr P , Winter MG , Butler BP , Huseby DL , Crawford RW , Russell JM , Bevins CL , Adams LG , Tsolis RM , et al. Gut inflammation provides a respiratory electron acceptor for salmonella. Nature. 2010;467:426–429. doi: 10.1038/nature09415. 20864996 PMC2946174

[76] Lopez CA , Winter SE , Rivera-Chávez F , Xavier MN , Poon V , Nuccio S-P , Tsolis RM , Bäumler AJ . Phage-mediated acquisition of a type III secreted effector protein boosts growth of salmonella by nitrate respiration. mBio. 2012;3, 10.1128/mbio.00143-12. PMC3374392 22691391

[77] Miao EA , Alpuche-Aranda CM , Dors M , Clark AE , Bader MW , Miller SI , Aderem A . Cytoplasmic flagellin activates caspase-1 and secretion of interleukin 1beta via ipaf. Nat Immunol. 2006;7:569–575. doi: 10.1038/ni1344. 16648853

[78] Sun Y-H , Rolán HG , Tsolis RM . Injection of flagellin into the host cell cytosol by salmonella enterica serotype typhimurium. J Biol Chem. 2007;282:33897–33901. doi: 10.1074/jbc.C700181200. 17911114

[79] Rauch I , Deets KA , Ji DX , Moltke J von , Tenthorey JL , Lee AY , Philip NH , Ayres JS , Brodsky IE , Gronert K , et al. NAIP-NLRC4 inflammasomes coordinate intestinal epithelial cell expulsion with eicosanoid and IL-18 release via activation of Caspase-1 and -8. Immunity. 2017;46:649–659. doi: 10.1016/j.immuni.2017.03.016. 28410991 PMC5476318

[80] Sekirov I , Tam NM , Jogova M , Robertson ML , Li Y , Lupp C , Finlay BB . Antibiotic-induced perturbations of the intestinal microbiota alter host susceptibility to enteric infection. Infect Immun. 2008;76:4726–4736. doi: 10.1128/IAI.00319-08. 18678663 PMC2546810

[81] Pompilio A , Crocetta V , De Nicola S , Verginelli F , Fiscarelli E , Di Bonaventura G . Cooperative pathogenicity in cystic fibrosis: *Stenotrophomonas maltophilia* modulates pseudomonas aeruginosa virulence in mixed biofilm. Front Microbiol [Internet]. 2015;6. doi: 10.3389/fmicb.2015.00951. [cited 2026 May 17]. Available from: https://www.frontiersin.org/journals/microbiology/articles/10.3389/fmicb.2015.00951/full. PMC4584994 26441885

[82] Roscetto E , Vitiello L , Muoio R , Soriano AA , Iula VD , Vollaro A , Gregorio ED , Catania MR . In vitro interaction of *Stenotrophomonas maltophilia* with human monocyte-derived dendritic cells. Front Microbiol [Internet]. 2015;6. doi: 10.3389/fmicb.2015.00723. [cited 2026 May 17]. Available from: https://www.frontiersin.org/journals/microbiology/articles/10.3389/fmicb.2015.00723/full. PMC4504169 26236302

[83] Brooke JS . *Stenotrophomonas maltophilia*: an emerging global opportunistic pathogen. Clin Microbiol Rev. 2012;25:2–41. doi: 10.1128/CMR.00019-11. 22232370 PMC3255966

[84] Yero D , Huedo P , Conchillo-Solé O , Martínez-Servat S , Mamat U , Coves X , Llanas F , Roca I , Vila J , Schaible UE , et al. Genetic variants of the DSF quorum sensing system in *Stenotrophomonas maltophilia* influence virulence and resistance phenotypes among genotypically diverse clinical isolates. Front Microbiol. 2020;11:1160. doi: 10.3389/fmicb.2020.01160. [cited 2025 Dec 22]. Available from: https://www.frontiersin.org/journals/microbiology/articles/10.3389/fmicb.2020.01160/full. 32582100 PMC7283896

[85] Coves X , Bravo M , Huedo P , Conchillo-Solé Ò , Gómez A-C , Esteve-Codina A , Dabad M , Gut M , Daura X , Yero D , et al. A *Stenotrophomonas maltophilia* TetR-Like transcriptional regulator involved in fatty acid metabolism is controlled by quorum sensing signals. Appl Environ Microbiol. 2023;89:e00635-23. doi: 10.1128/aem.00635-23. 37272812 PMC10304680

[86] Huedo P , Yero D , Martínez-Servat S , Estibariz I , Planell R , Martínez P , Ruyra A , Roher N , Roca I , Vila J , et al. Two different rpf clusters distributed among a population of *Stenotrophomonas maltophilia* clinical strains display differential diffusible signal factor production and virulence regulation. J Bacteriol. 2014;196:2431–2442. doi: 10.1128/JB.01540-14. 24769700 PMC4054175

[87] Heckman KL , Pease LR . Gene splicing and mutagenesis by PCR-driven overlap extension. Nat Protoc. 2007;2:924–932. doi: 10.1038/nprot.2007.132. 17446874

[88] Donnesburg MS , Kaper JB . Construction of an eae deletion mutant of enteropathogenic escherichia coli by using a positive-selection suicide vector. Infect Immun. 1991;59(12):4310–4317. doi: 10.1128/iai.59.12.4310-4317.1991. 1937792 PMC259042

[89] Datsenko KA , Wanner BL . One-step inactivation of chromosomal genes in escherichia coli K-12 using PCR products. Proc Natl Acad Sci. 2000;97:6640–6645. doi: 10.1073/pnas.120163297. 10829079 PMC18686

[90] Cherepanov PP , Wackernagel W . Gene disruption in escherichia coli: TcR and KmR cassettes with the option of flp-catalyzed excision of the antibiotic-resistance determinant. Gene. 1995;158:9–14. doi: 10.1016/0378-1119(95)00193-A. 7789817

[91] Sternberg NL , Maurer R . [2] bacteriophage-mediated generalized transduction in escherichia coli and salmonella typhimurium. In Methods Enzymol. 1991;204, pp. 18–43 Academic Press. Available from: https://www.sciencedirect.com/science/article/pii/0076687991040048. 1943777 10.1016/0076-6879(91)04004-8

[92] Fraser TA , Bell MG , Harris PNA , Bell SC , Bergh H , Nguyen T-K , Kidd TJ , Nimmo GR , Sarovich DS , Price EP . Quantitative real-time PCR assay for the rapid identification of the intrinsically multidrug-resistant bacterial pathogen *Stenotrophomonas maltophilia* . Microb Genom. 2019;5:e000307. doi: 10.1099/mgen.0.000307. 31617838 PMC6861864

[93] Folch J , Lees M , Stanley GHS . A SIMPLE METHOD FOR THE ISOLATION AND PURIFICATION OF TOTAL LIPIDES FROM ANIMAL TISSUES. J Biol Chem. 1957;226:497–509. doi: 10.1016/S0021-9258(18)64849-5. 13428781

[94] Livak KJ , Schmittgen TD . Analysis of relative gene expression data using real-time quantitative PCR and the 2−δδCT method. Methods. 2001;25:402–408. doi: 10.1006/meth.2001.1262. 11846609

[95] Hung C-C , Eade CR , Betteken MI , Pavinski Bitar PD , Handley EM , Nugent SL , Chowdhury R , Altier C . Salmonella invasion is controlled through the secondary structure of the hilD transcript. PLoS Pathog. 2019;15:e1007700. doi: 10.1371/journal.ppat.1007700. 31017982 PMC6502421

