# Supplementary material for: Salmonella exploits a quorum-sensing family signal of the gut commensal Stenotrophomonas maltophilia to facilitate its colonization
Source: Gut Microbes. 2026 Jul 8;18(1):2699455. doi: 10.1080/19490976.2026.2699455 (PMC13353783; doi:10.1080/19490976.2026.2699455)
Supplement: Supplementary Material — Upload Final Supplementary Figures 06112026.docx [file KGMI_A_2699455_SM2911.docx]

Title: ***Salmonella*exploits a quorum-sensing family signal of the gut commensal *Stenotrophomonas maltophilia* to facilitate its colonization.**

Authors: Rimi Chowdhury*^a^, Erick M. Bosire^b^, Lindsay R. Wolverton^a^, Paulina D. Pavinski Bitar^b^, Katherine E. Bell^b^, Ivan Keresztes^c^, Rory C. Chien^b^, Craig Altier^*,b^.

^a^ Department of Microbiology, College of Arts and Science, Miami University, Oxford OH 45056.

^b^ Department of Population Medicine and Diagnostic Sciences, College of Veterinary Medicine, Cornell University, Ithaca NY 14853.

^c^ Department of Chemistry and Chemical Biology, Cornell University, Ithaca NY 14853.

Correspondence: rchowdhury@miamioh.edu and altier@cornell.edu

**Supplementary Figures and Legends:**

**S1**

**
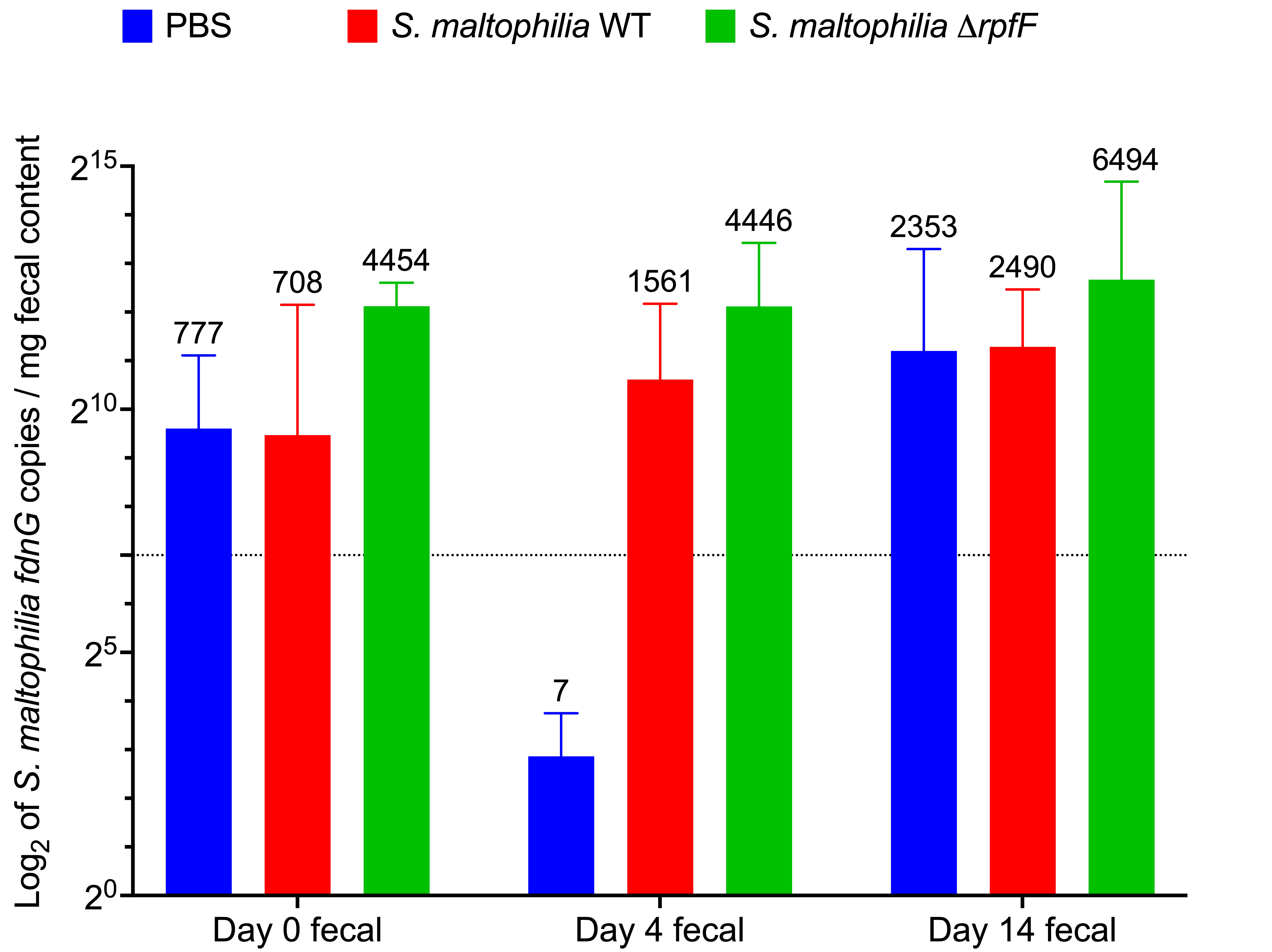
**

**Figure S1. Detection of *S. maltophilia* by RT-qPCR in murine fecal contents.**

Fecal contents were collected from groups of 129X1/SvJ mice (n=5) before (Day 0) and after treatment with PBS or *S. maltophilia* wild type or Δ*rpfF*. Total RNA was extracted and *S. maltophilia* *fdnG* copies were quantified by qPCR using equal amount of template and normalized per mg fecal content. Data show mean ± SD of five fecal replicates each having three technical replicates (total n = 15).

**S2**

**
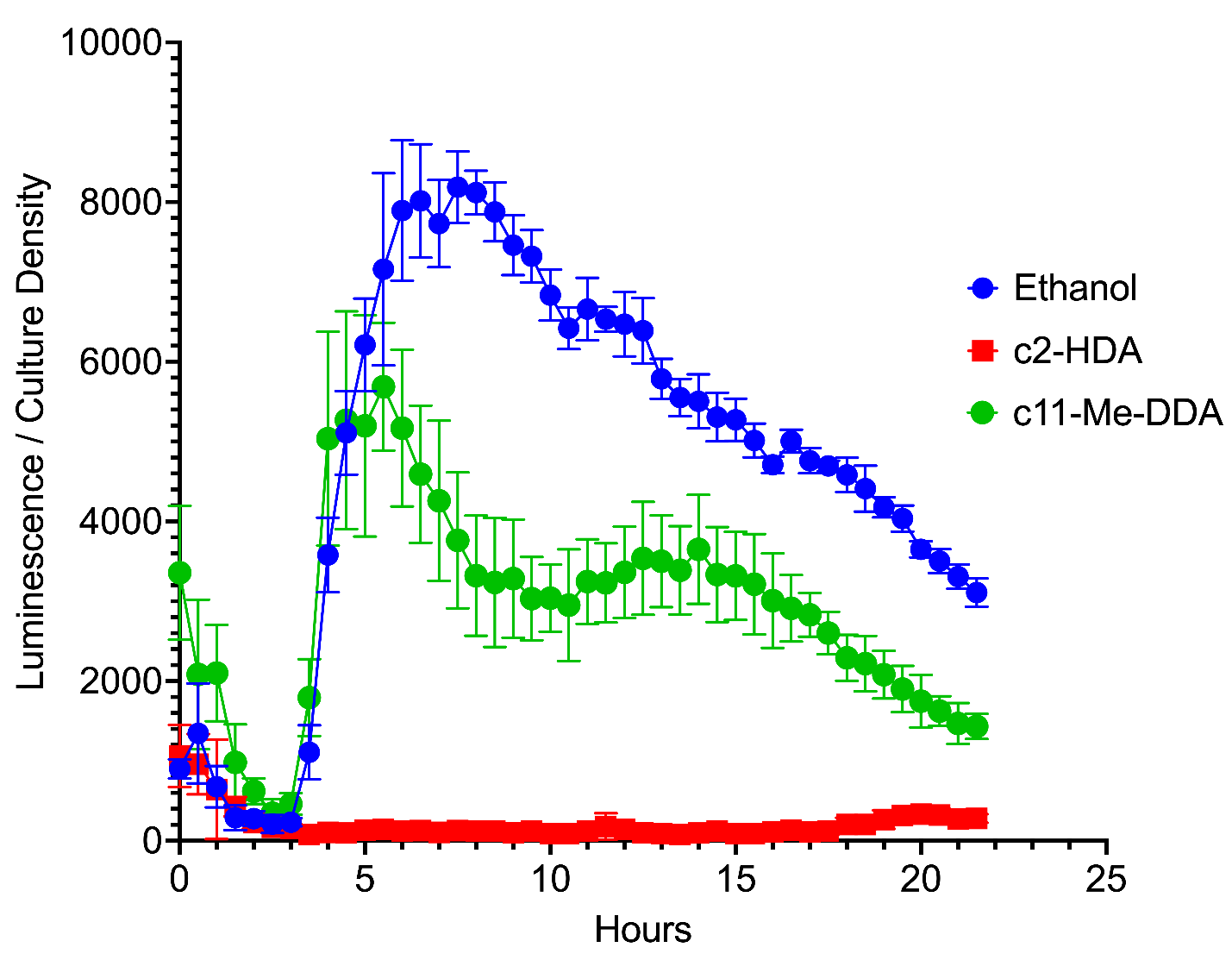
**

**Figure S2. Effect of DSF *cis*-2 11-methyl dodecenoic acid on *Salmonella* virulence.**

Effect of fatty acids on STm virulence. Fatty acids c2-HDA (2 µM) or c2-Me-DDA (20 µM) were added to growing STm cultures having the luminescence reporter plasmid *philA-luxCDABE*. OD600 and luminescence were monitored for 24 hours and measurements were recorded every half-hour. Curves show mean ± SD (n = 5).

**S3**

**
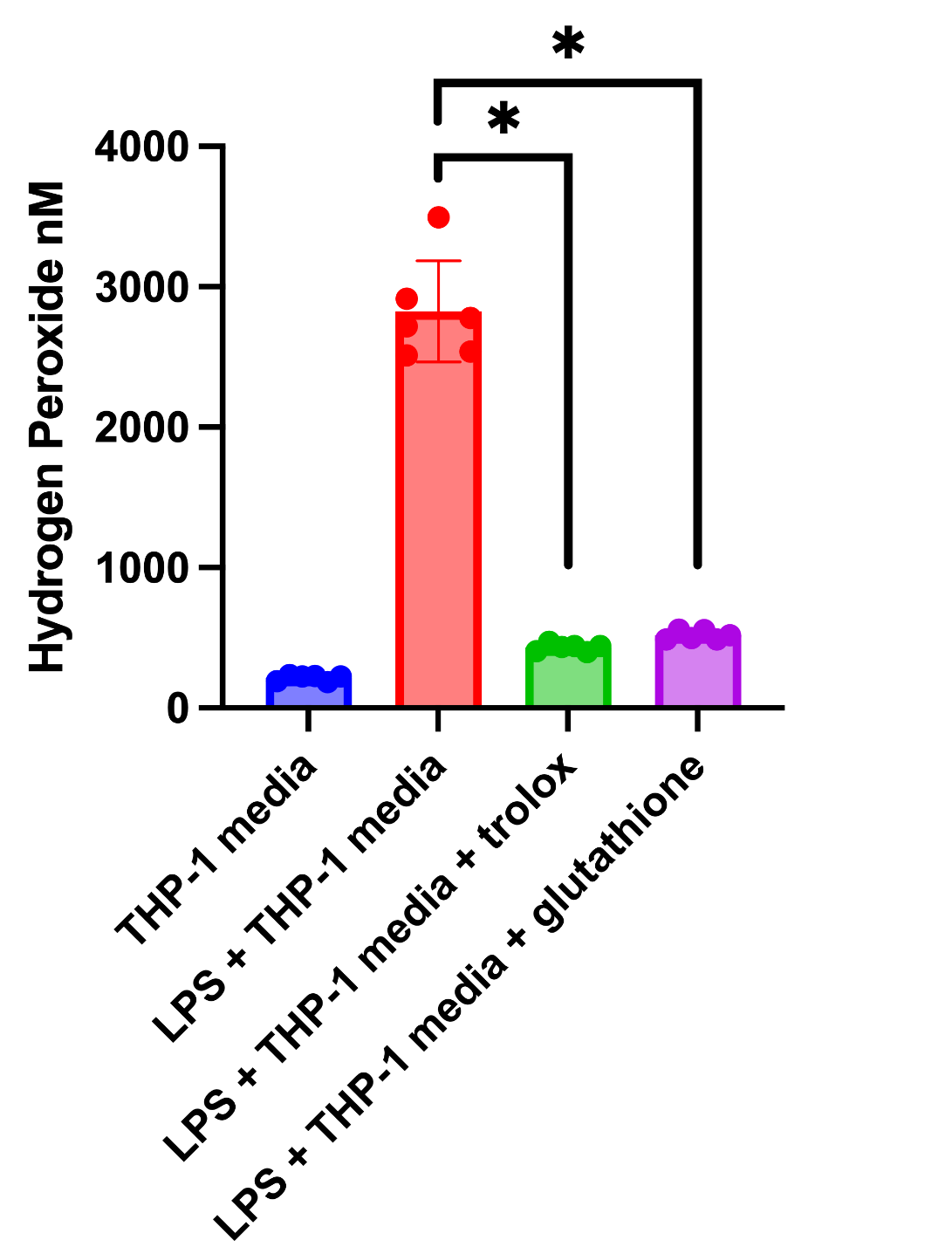
**

**S4**

**
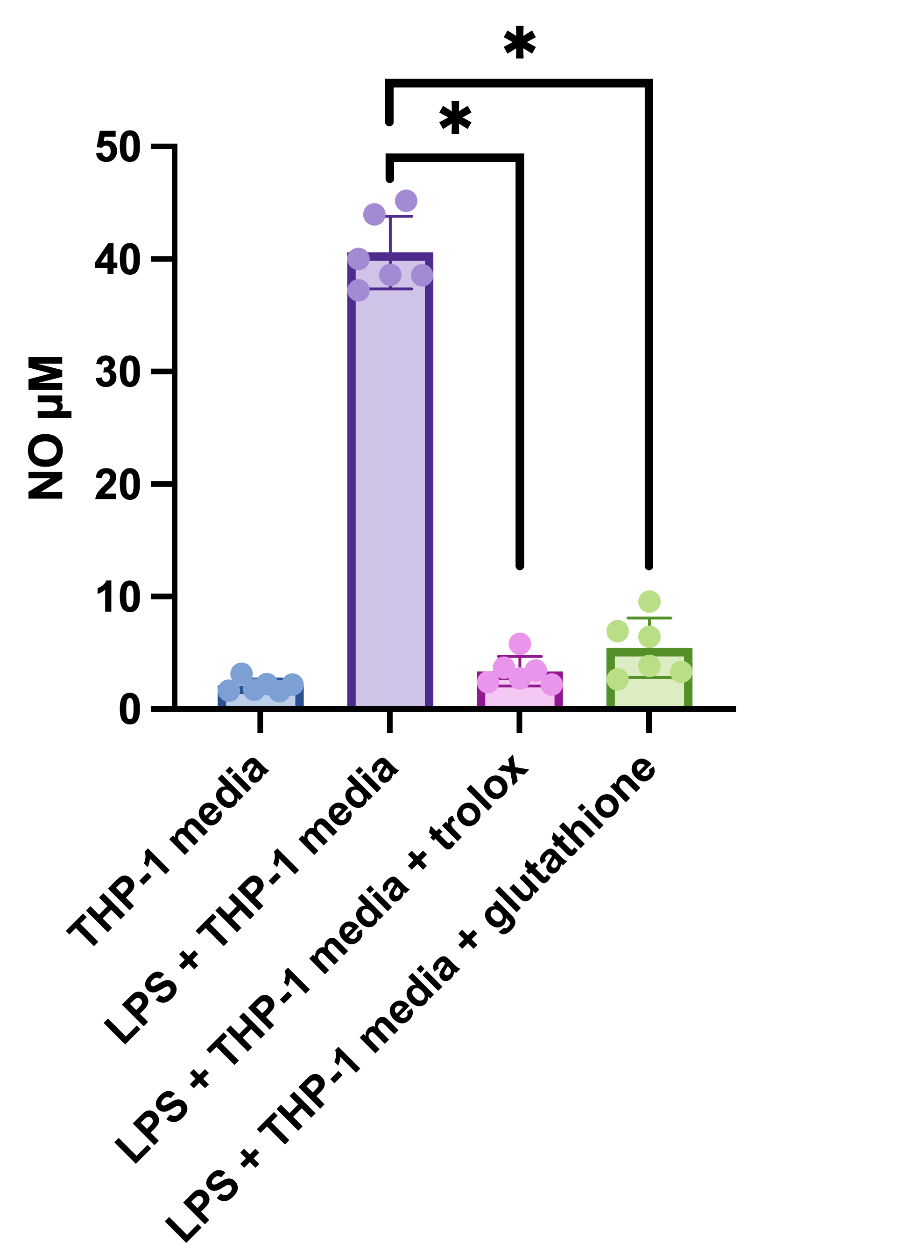
**

**Figure S3-4: Quantification of ROS and RNS and the effect of addition of the antioxidant Trolox.**

Cell supernatants of THP-1 macrophages stimulated with LPS with or without Trolox or glutathione treatment were collected. Amplex Red (**S3**) and Greiss reagent assay (**S4**) were performed to quantify ROS (HP) and RNS ((Nitric oxide, NO) respectively, and the color was read spectrophotometrically. Concentration of HP and NO were determined by a standard curve. Bars show HP concentration in nM ± SD (n = 6) and NO concentration in µM ± SD (n = 5) and. Differences were calculated by Mann-Whitney test. *, P <0.05.


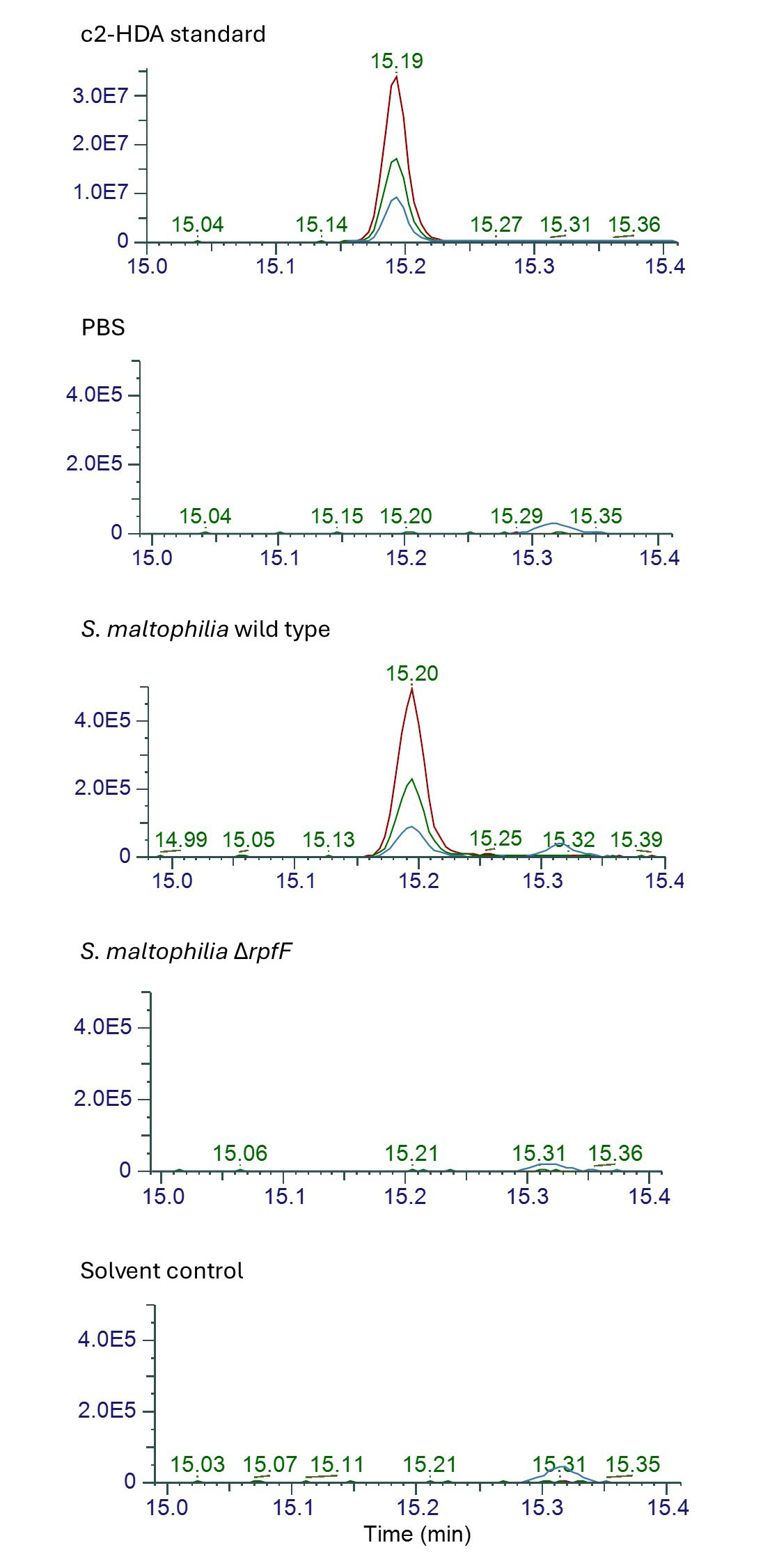


1

2

3

4

5

**Figure S5: Detection of c2-HDA in murine colon contents of *S. maltophilia* mouse model.**

Extracted ion chromatograms (EICs) of fatty acid samples extracted from murine colon contents (n=5), highlighting a key DSF peak at a retention time of ~15.20 minutes. Overlayed EICs are shown for *m/z* 113.0591 (red), 81.0331 (green), and 171.1370 (blue) ± 5 ppm. 1. standard c2-HDA, 5. Solvent control; fatty acids extracted from colon contents of the mice inoculated with (2) PBS or (3) *S. maltophilia* wild type or (4) *S. maltophilia* Δ*rpfF.*

**S6**


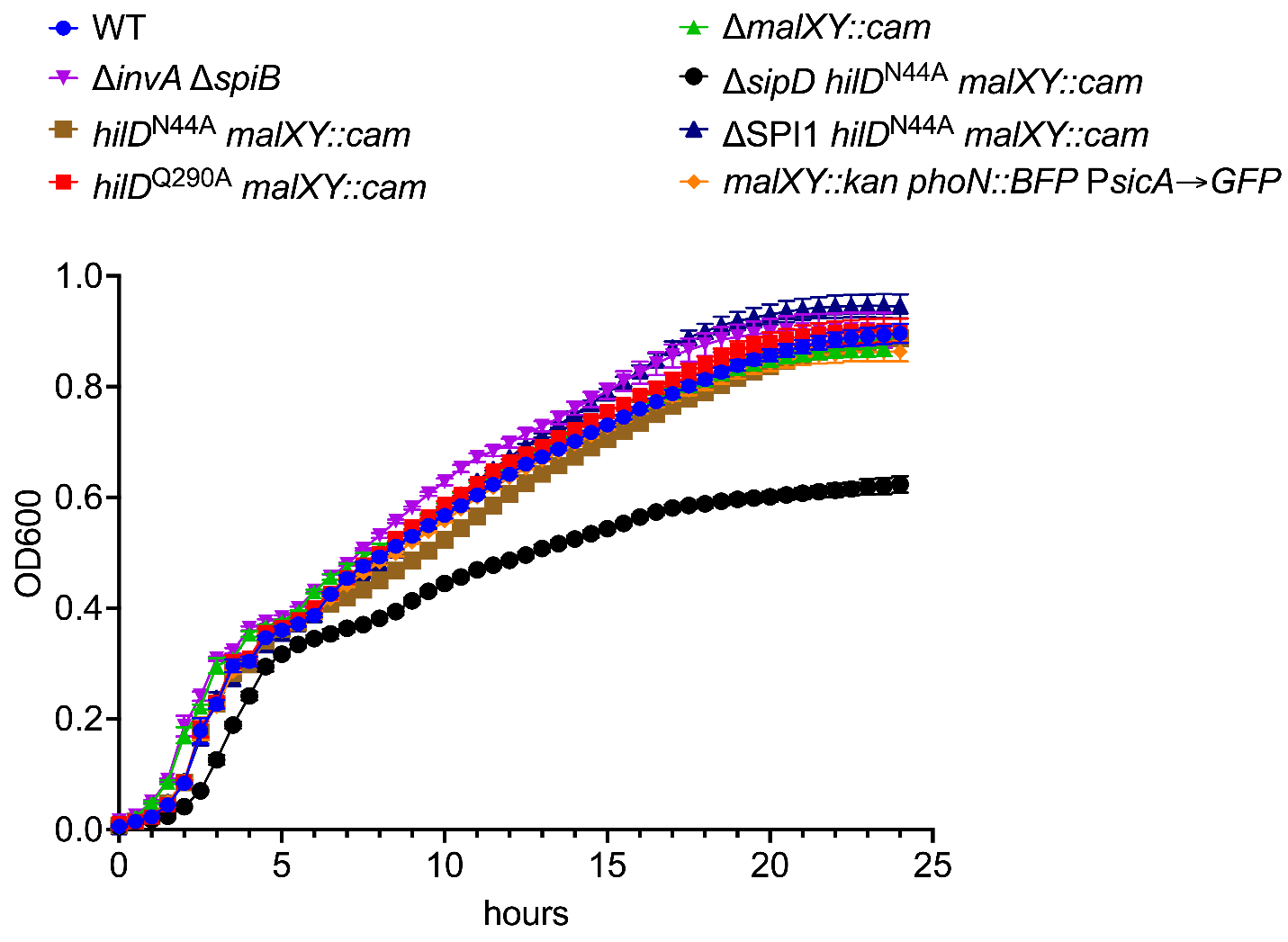


**Figure S6. Effect of various genetic mutations on *Salmonella* growth rate.**

Various STm cultures having different genetic mutations were grown and OD600 was monitored for 24 hours and measurements were recorded every half-hour. Curves show the mean ± SD (n = 5).

**S7**

**A B C**


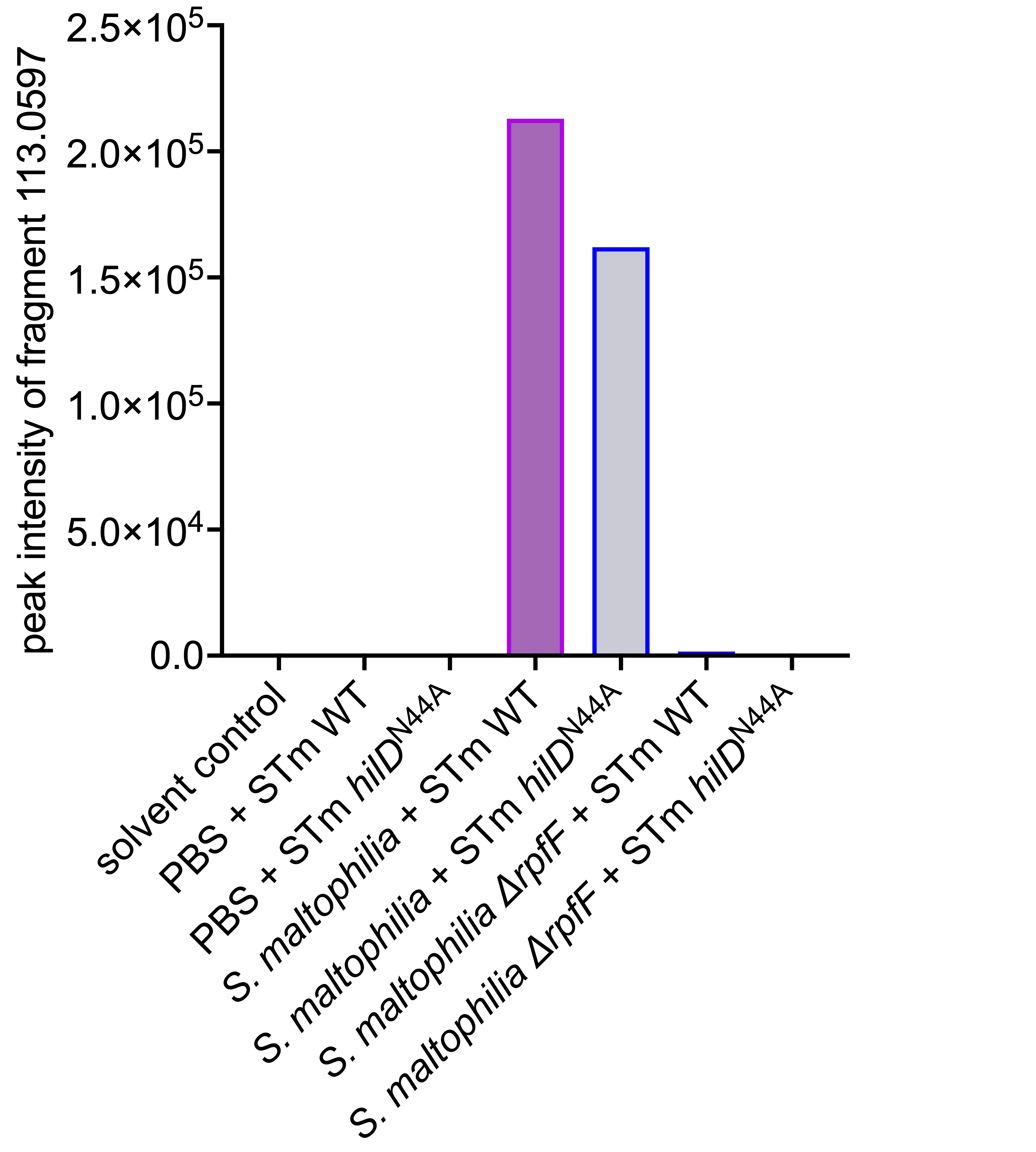

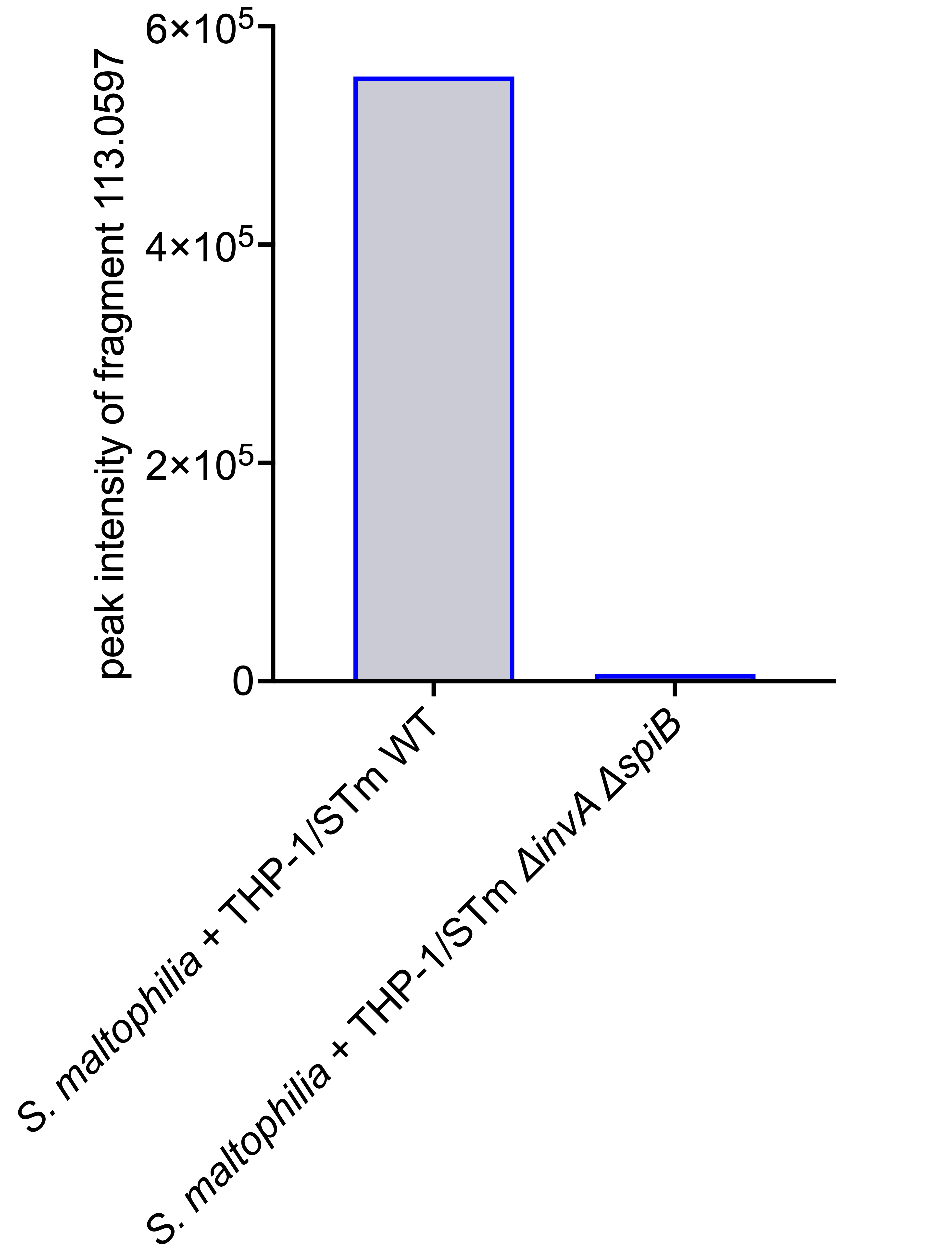

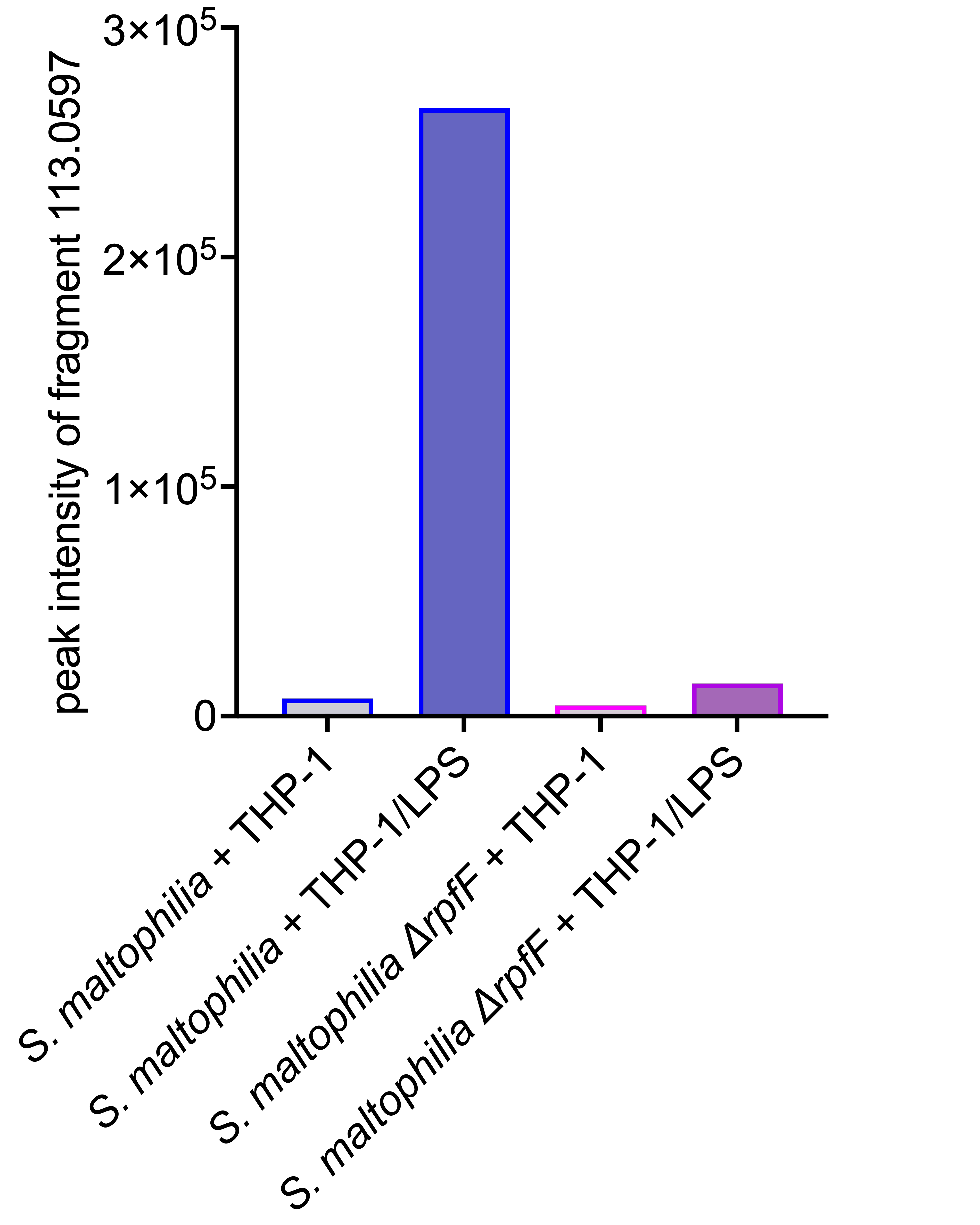


**Figure S7**. Bar graphs of the quantification of the 113.0591 peak of the EICs shown in Fig 4 (**A**), Fig 5 (**B**) and Fig 6 (**C**).
