# Supplementary material for: Salmonella exploits a quorum-sensing family signal of the gut commensal Stenotrophomonas maltophilia to facilitate its colonization
Source: Gut Microbes. 2026 Jul 8;18(1):2699455. doi: 10.1080/19490976.2026.2699455 (PMC13353783; doi:10.1080/19490976.2026.2699455)
Supplement: Supplementary Material — Supplementary Methods Mar 1.docx [file KGMI_A_2699455_SM2910.docx]

Title: ***Salmonella*exploits a quorum-sensing signal of the gut commensal *Stenotrophomonas maltophilia* to facilitate its colonization.**

Authors: Rimi Chowdhury*^a^, Erick M. Bosire^b^, Lindsay R. Wolverton^a^, Paulina D. Pavinski Bitar^b^, Katherine E. Bell^b^, Ivan Keresztes^c^, Rory C. Chien^b^, Craig Altier^*,b^.

^a^ Department of Microbiology, College of Arts and Science, Miami University, Oxford OH 45056.

^b^ Department of Population Medicine and Diagnostic Sciences, College of Veterinary Medicine, Cornell University, Ithaca NY 14853.

^c^ Department of Chemistry and Chemical Biology, Cornell University, Ithaca NY 14853.

Correspondence: rchowdhury@miamioh.edu and altier@cornell.edu

**Supplementary methods**

**Histology of murine intestine and scoring:**

These experiments were performed on *Salmonella*-infected *S. maltophilia*-colonized mice. On day 5, after euthanasia, distal ileums, cecums and proximal colons of mice were collected and fixed in 10% neutral-buffered formalin, embedded in paraffin, sectioned, and stained with hematoxylin and eosin (H&E). Histopathologic changes of the cecum were evaluated blindly by a board-certified pathologist (RCC) and scored with a semiquantitative scale modified from Barthel et al. Evaluated criteria include the degree of submucosal edema, severity of neutrophilic infiltration, number of goblet cells, and epithelial integrity. The details of the scoring process are described below:

- Submucosal edema. The degree of submucosal edema was scored as follows: 0 = no significant changes; 1 = mild edema (the submucosa accounts for <50% of the intestinal wall [tunica muscularis to epithelium]); 2 = moderate edema (the submucosa accounts for 50%-80% of the intestinal wall; 3 = profound edema (the submucosa accounts for >80% of the intestinal wall).
- Neutrophilic infiltration in the mucosa. The number of neutrophils in the lamina propria were counted in ten 400x fields (2.37 mm^2^) and was scored as follows: 0 = less than 100 neutrophils; 1 = 100 to 300 neutrophils; 2 = 300 to 1000 neutrophils; 3 = 1000 to 1800 neutrophils; 4 = more than 1800 neutrophils.
- Goblet cells. Mucosal remodeling and reduction of goblet cells were observed secondary to mucosal damage and acute inflammation in the cecum. The number of goblet cells in the cecal crypts were counted in ten 400x fields (2.37 mm^2^) and was scored as follows: 0 = more than 500 goblet cells; 1 = 200 to 500 goblet cells; 2 = 20 to 200 goblet cells; 3 = less than 20 goblet cells.
- Mucosal epithelial integrity. The epithelial lining of the cecal mucosa was examined in ten 400x fields (2.37 mm^2^) and was scored as follows: 0 = no detectable pathological changes; 1 = epithelial attenuation (flattening of the lining epithelium); 2 = mucosal erosion (evidence of epithelial loss but the basement membrane remained intact); 3 = mucosal ulceration (complete loss of the lining epithelium with exposure of the lamina propria).
